# Multiple refugia from penultimate glaciations in East Asia demonstrated by phylogeography and ecological modelling of an insect pest

**DOI:** 10.1186/s12862-018-1269-z

**Published:** 2018-10-11

**Authors:** Wei Song, Li-Jun Cao, Bing-Yan Li, Ya-Jun Gong, Ary Anthony Hoffmann, Shu-Jun Wei

**Affiliations:** 1 Institute of Plant and Environmental Protection, Beijing Academy of Agriculture and Forestry Sciences, 9 Shuguanghuayuan Middle Road, Haidian District, Beijing, 100097 China; 2 College of Forestry, Beijing Forestry University, Beijing, 100083 China; 3 School of BioSciences, Bio21 Institute, The University of Melbourne, Melbourne, VIC 3010 Australia

**Keywords:** Approximate Bayesian computation, Eastern Asia, Endemic population, Glacial refugia, Quaternary climate

## Abstract

**Background:**

Refugial populations in Quaternary glaciations are critical to understanding the evolutionary history and climatic interactions of many extant species. Compared with the well-studied areas of Europe and Northern America, refugia of species in eastern Asia remain largely unknown. Here, we investigated the phylogeographic history of a globally important insect pest, the oriental fruit moth *Grapholita molesta*, in its native range of China.

**Results:**

Genetic structure analyses unveiled three distinct groups and a set of populations with admixture. Approximate Bayesian Computation (ABC) analyses support range expansion of this moth from southwest groups of Yunnan and Sichuan to northern and eastern China. A set of admixed populations was found around these two ancestral groups. This pattern of genetic structure points to two refugia located in the Yunnan region and Sichuan Basin. The split of the two refugia was dated to 329.2 thousand years ago in the penultimate glacial period. One of the lineages was exclusively found around the Sichuan Basin, indicating the formation of endemic populations in this refugium. Ecological niche model analysis suggested a shrinking distribution from the LIG period to the MID period in the Sichuan lineage but a wide and stable distribution in the other lineage.

**Conclusions:**

Our results for the first time suggest that Yunnan and Sichuan jointly served as two large-scale refugia in eastern Asia in Quaternary glaciations, helping to maintain genetic diversity overall.

**Electronic supplementary material:**

The online version of this article (10.1186/s12862-018-1269-z) contains supplementary material, which is available to authorized users.

## Background

Quaternary climatic oscillations (2.6 million years ago–present) have profoundly impacted the current distribution and genetic diversity of extant species [1, 2]. Species contracted to refugial areas during cold glaciation periods, and then expanded their distribution ranges on warm interglacial periods [1, 2]. Under glacial cycling, refugia play an important role in maintaining genetic diversity and providing sources of admixture in postglacial colonization [3, 4]. Identifying refugial populations sheds light on the influence of Quaternary climate in current distributions and may also help in predicting species responses to future climatic change [5–8].

Identification of refugia has always been challenging [5, 9]. Responses of species to climatic changes may be different due to their unique biology and historical distributions [10]. This leads to variable genetic imprints when tracing the evolutionary history of species. Refugial populations usually possess higher genetic diversity than the derived populations, however, high genetic diversity can be found in non-refugial regions due to hybridization in postglacial colonization [5] and high gene flow from refuge areas [11]. Human-mediated dispersal can reshape the genetic structure of animal populations and superimpose new signatures on existing natural phylogeographical patterns [12]. Patterns of genetic structure among species can be different due to their dispersal ability [13]. Species with low dispersal ability can be used to infer refugia in a local region while species with higher dispersal ability can be used to infer refugia on a larger scale.

Recent developments around hypothesis-based approaches, such as Approximate Bayesian Computation (ABC) [14], and the integration of species distribution models [15], can help to resolve complex scenarios around phylogeographical patterns. An increasing number of phylogeographical studies have successfully identified refugial populations using these methods when combined with a deep understanding of population differentiation [5–8, 16].

The impacts of Quaternary climate on the distribution and genetic diversity of extant species have been well studied in Europe and North America [1, 2, 17]. Multiple refugia were identified in regions as part of these studies [9, 18]. Most species in regions considered in these studies were influenced by the Last Glacial Maximum (LGM) dated to 27–19 thousand years ago (kya) [18]. In Europe, Mediterranean peninsulas acted as glacial refugia and as sources of recolonization [2] while recent studies have also identified multiple refugia for species in northern Europe [6].

Unlike Europe and North America, the climate was warmer in eastern Asia with less area covered by ice sheets in the LGM [17, 19]. Patterns of genetic diversity influenced by Quaternary climate may therefore be different in eastern Asia. On a spatial scale, refugia have been traced to southern areas [3, 20] and to northern areas [21]. The Qinghai-Tibetan Plateau (QTP) is the hotspot for many phylogenetic and phylogeographic studies in East Asia [22, 23]. The uplift of QTP leads to numerous endemic species in this area [24]. Multiple microrefugia were identified in QTP and its adjacent areas for many endemic species [22, 25, 26]. Other studies focused on regional areas, such as northeastern China [21, 27, 28] and southern China [20, 29] and refugia were identified within these areas. There is a lack of study on wider range-scale identification of Quaternary climate on species evolutionary history in East Asia [30–32], which might be caused by the limited distribution of the species used in previous studies. On a temporal scale, divergence times of populations appear to be variable, dating to Late Pliocene to Early Pleistocene [33], the penultimate glacial period (about 170 kya) [34], and the LGM [35]. The repeated influence by Quaternary climate oscillations made the postglacial history of species complicated in east Asia. Range expansion and inter−/postglacial recolonization were reported in species of east Asia [36–38], however, limited population admixture with multiple refugia existed in some other species [29]. Thus, species in eastern Asia might be survived in single or multiple refugia and impacted by different glacial periods of the Quaternary, although the evidence remains relatively limited.

The oriental fruit moth (OFM), *Grapholita molesta* (Busck, 1916) (Lepidoptera: Tortricidae) is a major pest of stone and pome fruit, especially species of Rosaceae [39], inflicting severe damage to orchards [39]. Larvae of the OFM cause damage by boring into twigs as well as fruits. Historical and population level studies traced the native range of this species to East Asia; it spread into other stone-fruit growing regions, including the Middle East, Europe, Africa, South and North America, New Zealand and Australia, from eastern Asia in the last century [39, 40]. Our recent study using population genetic approach revealed that southwest China around Yunnan is the area where OFM originated [41]. A weak but significant genetic structure on a continental scale was found on the global studies of the OFM [40]. Strong population genetic structure with isolation by distance was found in its native range of China; the estimated divergence time between the two haplotype lineages fall within the Pleistocene [41]. In small sale of Brazil, geographic distance was defined as the main factor affecting genetic structure and gene flow in the invasive populations [42]. These studies showed that the genetic structure of the OFM was strongly influenced by geographical isolation [41, 42]. In introduced areas of South Africa and Italy, no pattern of isolation by distance was found within populations of the OFM, indicating that active and passive dispersal associated with accidental anthropogenic displacements helped to expand their geographical range [43, 44]. Structured populations from different orchards within an area indicated a selective host switch occurred in certain segments of the population [45]. The high level of genetic differentiation, wide distribution and evidence of dispersal make OFM a promising model species to identify refugia and test scenarios of past climate in shaping genetic patterns in eastern Asia.

In southwestern China near Yunnan province, a highly diverged mitochondrial lineage was identified in populations from the Sichuan Basin, indicating this area is a putative refugium during glaciations for OFM [41]. Thus, both the original area of Yunnan and Sichuan Basin may server as refugia of the OFM during Quaternary. Because that only single representative populations from Yunnan and Sichuan have been considered so far [41], patterns of genetic differentiation in its native region remain unclear, which limits the identification of refugia of OFM.

In this study, we explored genetic diversity and population structure within and around the original area of OFM in southwestern China. We chose 12 microsatellite loci developed from genome-scale analysis in and two mitochondrial genes in the current study because both sets of genetic markers can reveal genetic differentiation at small and large scales [40, 41, 44]. We hypothesized that there were multiple refugia located in the native range of OFM in southwestern China. By densely sampling the native range, we aimed to reveal refugia of OFM in the Quaternary and the impact of climate oscillations on population patterns. Specifically, we verified whether the Sichuan Basin served as a refugium and its relationship to the native range in Yunnan and surrounding areas. By examining the population history of a widespread insect species in its native range of East Asia, we also aimed to understand the impact of Quaternary climate on species history on a large spatial scale and long temporal scale.

## Results

### Power of microsatellite markers and population genetic diversity

POWSIM analysis showed that our microsatellite loci are sufficient to provide a 96% probability of detecting an *F*_*ST*_ as low as 0.0025 for all populations and 100% probability of detecting an *F*_*ST*_ as low as 0.005, indicating that the markers genotyped in our study had strong statistical power to investigate population differentiation in OFM (Additional file 1: Table S1).

Based on microsatellite loci, the expected heterozygosity ranged from 0.393 to 0.696 with an average of 0.560 for individual population (Table 1). The observed heterozygosity was significantly lower than those corresponding *H*_*e*_, falling between 0.256 and 0.363 with an average of 0.313. The average number of alleles was the smallest in the GDGZ population (Table 1). There was a relatively high null allele frequency in each population, ranging from 0.099 to 0.226, and the null allele frequency among the 12 loci was between 0.015 and 0.246. The inbreeding coefficients of each geographical population are high (Table 1), possibly related to sampling of OFM (although individuals were sampled from different trees). However high *F*_*IS*_ also results from null alleles. Hardy–Weinberg tests show that 6 out of 12 loci and most of the individual population significantly deviated.

**Table 1:** Genetic diversity of the *Grapholita molesta* populations

Group	Population	*He*	*Ho*	*A*_*R*_	NAF	*F*_*IS*_	*S*	*Hd*	*Pi*
YN	YNPE	0.393	0.256	2.793	0.113	0.357	18	0.426	0.002
YN	YNBS	0.559	0.354	3.444	0.135	0.372	18	0.757	0.002
YN	YNHH	0.513	0.362	3.156	0.099	0.301	2	0.368	0.000
YN	YNQJ	0.546	0.348	3.550	0.129	0.369	1	0.833	0.000
YN	YNKM	0.528	0.356	3.540	0.128	0.331	4	0.678	0.001
SC	SCCD	0.683	0.287	5.129	0.226	0.586	11	0.891	0.003
SC	SCNC	0.696	0.363	5.191	0.194	0.484	24	0.790	0.003
SC	SCBZ	0.627	0.359	4.733	0.154	0.434	19	0.706	0.003
SC	SCGY	0.661	0.344	4.979	0.183	0.488	29	0.944	0.006
AM	GDGZ	0.451	0.207	2.819	0.156	0.547	6	0.377	0.001
AM	GXNN	0.552	0.346	3.550	0.131	0.38	11	0.726	0.001
AM	GZGY	0.623	0.257	4.532	0.216	0.594	26	0.982	0.005
AM	GSTS	0.562	0.340	3.832	0.147	0.402	16	0.881	0.004
AM	QHHD	0.567	0.309	3.696	0.166	0.463	12	0.236	0.001
NE	HNCS	0.561	0.294	3.676	0.154	0.488	21	0.894	0.004
NE	FJND	0.562	0.308	4.144	0.154	0.458	10	0.889	0.002
NE	ZJNB	0.602	0.269	4.108	0.196	0.56	11	0.771	0.001
NE	SXYA	0.566	0.294	3.958	0.165	0.486	18	0.841	0.002
NE	SDQD	0.499	0.279	3.589	0.136	0.449	10	0.810	0.001
NE	BJPG	0.519	0.333	3.882	0.124	0.363	11	0.830	0.002
NE	LNSY	0.489	0.301	3.525	0.120	0.390	8	0.685	0.001

*He* expected heterozygosity, *Ho* observed heterozygosity, *A*_*R*_ allele richness, *NAF* null allele frequency, *F*_*IS*_ inbreeding coefficients, *S* number of polymorphic (segregating) sites, *Hd* haplotype diversity; *Pi*, nucleotide diversity. The underlined numbers are the top five values of *A*_*R*_ and *Hd* in all populations

Based on the mitochondrial marker, 109 haplotypes were observed from the 491 combined genes. The haplotype diversity was 0.9 evaluated from the combined mitochondrial genes and among the individual population it was also significantly high, ranged from 0.236 for QHHD population to 0.982 for GZGY population (Table 1).

### Population genetic structure

Among the 21 populations, high pairwise *F*_*ST*_ values were found between populations whenever null alleles were considered (Table 2).

**Table 2:** *F*_*ST*_ values calculated in FREENA based on microsatellite loci

Population	BJPG	FJND	GDGZ	GSTS	GXNN	GZGY	HNCS	LNSY	QHHD	SCBZ	SCCD	SCGY	SCNC	SDQD	SXYA	YNBS	YNHH	YNKM	YNPE	YNQJ	ZJNB
BJPG	–	0.035	0.193	0.068	0.081	0.069	0.021	0	0.045	0.065	0.078	0.083	0.095	0.025	0.02	0.108	0.135	0.088	0.213	0.131	0.027
FJND	0.032	–	0.175	0.054	0.073	0.06	0.014	0.042	0.053	0.067	0.065	0.075	0.081	0.039	0.054	0.112	0.106	0.11	0.197	0.14	0.031
GDGZ	0.218	0.193	–	0.194	0.192	0.126	0.155	0.181	0.16	0.165	0.164	0.164	0.181	0.206	0.17	0.157	0.19	0.164	0.2	0.168	0.154
GSTS	0.076	0.067	0.222	–	0.1	0.041	0.049	0.074	0.025	0.053	0.04	0.022	0.062	0.061	0.05	0.111	0.165	0.117	0.188	0.13	0.024
GXNN	0.082	0.078	0.211	0.108	–	0.052	0.087	0.092	0.109	0.091	0.085	0.089	0.084	0.122	0.122	0.128	0.16	0.13	0.228	0.14	0.083
GZGY	0.067	0.064	0.139	0.046	0.05	–	0.048	0.068	0.049	0.033	0.019	0.019	0.037	0.089	0.07	0.063	0.116	0.075	0.123	0.065	0.033
HNCS	0.034	0.015	0.175	0.058	0.099	0.051	–	0.038	0.036	0.054	0.058	0.05	0.067	0.019	0.021	0.091	0.106	0.086	0.176	0.124	0.016
LNSY	−0.005	0.044	0.208	0.091	0.097	0.07	0.054	–	0.061	0.075	0.082	0.086	0.105	0.039	0.044	0.108	0.134	0.098	0.195	0.138	0.042
QHHD	0.052	0.057	0.183	0.024	0.112	0.051	0.04	0.074	–	0.058	0.06	0.052	0.078	0.033	0.029	0.084	0.134	0.07	0.175	0.111	0.022
SCBZ	0.074	0.077	0.181	0.064	0.106	0.04	0.063	0.092	0.066	–	0.011	0.013	0.012	0.071	0.061	0.098	0.172	0.108	0.175	0.105	0.02
SCCD	0.076	0.068	0.176	0.039	0.087	0.022	0.053	0.088	0.069	0.01	–	0.004	0.01	0.085	0.07	0.09	0.16	0.113	0.176	0.109	0.019
SCGY	0.079	0.075	0.177	0.018	0.089	0.018	0.045	0.089	0.046	0.009	0	–	0.012	0.089	0.06	0.092	0.162	0.109	0.155	0.101	0.023
SCNC	0.097	0.083	0.195	0.063	0.094	0.046	0.067	0.115	0.078	0.007	0.011	0.011	–	0.101	0.08	0.11	0.175	0.124	0.191	0.122	0.035
SDQD	0.037	0.039	0.225	0.063	0.133	0.088	0.023	0.049	0.032	0.084	0.084	0.083	0.1	–	0.026	0.114	0.166	0.101	0.22	0.157	0.023
SXYA	0.014	0.057	0.191	0.054	0.124	0.074	0.029	0.039	0.028	0.067	0.074	0.062	0.083	0.02	–	0.083	0.13	0.09	0.191	0.118	0.018
YNBS	0.124	0.128	0.177	0.128	0.132	0.064	0.107	0.128	0.098	0.109	0.097	0.09	0.117	0.13	0.091	–	0.074	0.062	0.067	0.058	0.069
YNHH	0.148	0.114	0.218	0.186	0.156	0.108	0.124	0.155	0.15	0.182	0.158	0.16	0.177	0.185	0.143	0.089	–	0.098	0.098	0.073	0.142
YNKM	0.108	0.124	0.189	0.143	0.139	0.081	0.105	0.122	0.091	0.13	0.129	0.117	0.139	0.123	0.106	0.071	0.109	–	0.106	0.064	0.091
YNPE	0.236	0.216	0.229	0.215	0.236	0.119	0.193	0.231	0.196	0.19	0.179	0.157	0.197	0.246	0.21	0.066	0.109	0.113	–	0.054	0.178
YNQJ	0.149	0.161	0.2	0.156	0.151	0.068	0.146	0.163	0.134	0.124	0.122	0.108	0.136	0.183	0.139	0.07	0.076	0.079	0.058	–	0.108
ZJNB	0.027	0.035	0.169	0.026	0.087	0.037	0.018	0.043	0.028	0.028	0.025	0.022	0.041	0.017	0.012	0.076	0.154	0.113	0.194	0.131	–

The upper matrix is *F*_*ST*_ values calculated in FREENA using ENA model; the lower is *F*_*ST*_ values calculated in FREENA without ENA model

**Fig. 1:**
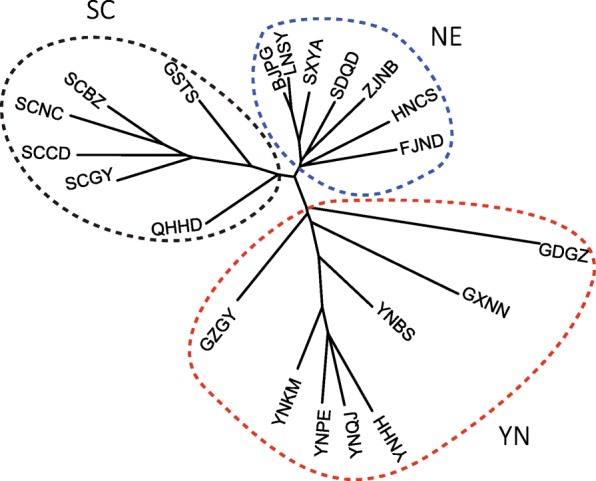
Phylogenetic relationships among the populations of *Grapholita molesta* based on microsatellite loci. The three circles marked by a dashed line show the three groups identified in the populations of *G. molesta*, i.e., Yunnan group (YN), Sichuan group (SC) and group in eastern and northern regions (NE)

Based on combined mitochondrial genes and/or microsatellites, the POPTREE2 analysis generated the same topology, in which populations of OFM were divided into three genetic lineages. One was formed by populations from Yunnan and three populations around Yunnan (YN), another was formed by populations from Sichuan and two surrounding populations (SC), and the third was formed by seven populations from the northern and eastern region (NE) (Fig. 1).

**Fig. 2:**
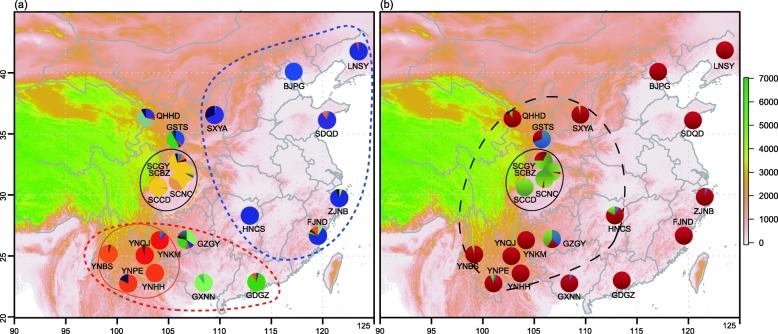
Collection sites, BAPS analysis of the population structure based on microsatellite loci (**a**) and mitochondrial genes (**b**). The three circles in figure (**a**) marked by a dashed line show the three groups identified in the populations of *G. molesta*: the orange circles show the Yunnan group (YN), the yellow ones show the Sichuan group (SC) while the blue circles show the other group in eastern and northern regions (NE). The two solid line circles in figure (**a**) display the core populations in YN and SC groups. The dash line circle in figure (**b**) shows the SC groups, while the solid line circle indicates core populations. The color bar shows the altitude (m). Codes of populations are listed in Table 3

BAPS analysis using microsatellites identified a similar genetic pattern. Different clusters from those in the core populations of Yunnan and Sichuan were identified in surrounding populations (Fig. 2a). BAPS analysis based on mitochondrial genes revealed three clusters, the major one was widely distributed in populations from Yunnan and northern and eastern regions; the second one was mainly distributed in population from Sichuan Basin and the other one was mainly distributed in populations surrounding Sichuan Basin (Fig. 2b).

**Fig. 3:**
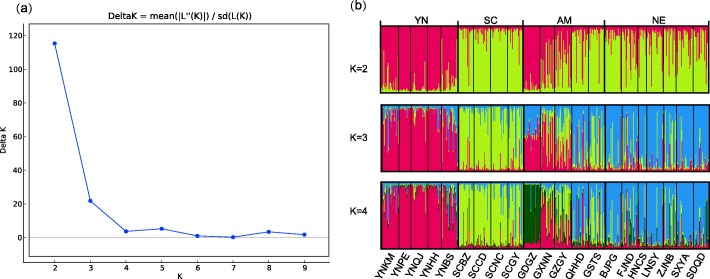
Delta K (**a**) and population genetic structure (**b**) of *Grapholita molesta* analyzed by STRUCTURE. Codes for the populations and groups are given in Table 3. YN, Yunnan group; SC, Sichuan group; NE, northern and eastern group; AM, set of populations with admixture

STRUCTURE analysis indicated the optimal number of clusters was two (Fig. 3a). Increasing the number of clusters showed a clear pattern with three major clusters (Yunnan, Sichuan and NE) and a group of populations with admixture (AM in Fig. 3b).

**Fig. 4:**
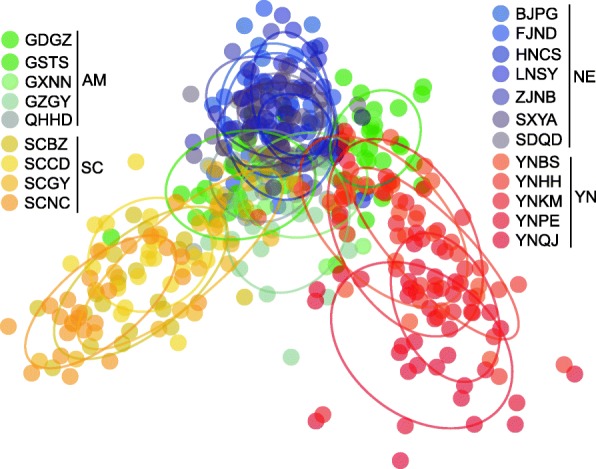
Discriminant Analysis of Principal Components (DAPC) in populations of *Grapholita molesta*. Codes for the populations and groups are given in Table 3. YN, Yunnan group; SC, Sichuan group; NE, northern and eastern group; AM, set of populations with admixture

In DAPC analysis, an inverted V-shaped pattern for the distribution of populations was evident. Populations from Yunnan and Sichuan located at two tips. Populations from NE and AM connected the two groups at the top position (Fig. 4), suggesting these populations are derived from Yunnan and Sichuan.

Finally, we tested isolation by distance and correlation between two sets of genetic markers. Based on microsatellites, there was significant IBD in populations of OFM (*r* = 0.311, *p* = 0.002), however, when based on mitochondrial genes, no IBD was present (*r* = 0.018, *p* = 0.417). Incongruent between microsatellites and mitochondrial genes in IBD was confirmed by no significant correlation between pairwise *F*_*ST*_ values calculated from them. Outliers of IBD plots based on mitochondrial genes were found in population pairs involving Sichuan and admixture populations. When we excluded those populations, IBD was present in an analysis based on mitochondrial genes (*r* = 0.422, *p* = 0.003) and significant correlation was found between pairwise *F*_*ST*_ values calculated from two sets of markers (*r* = 0.4, *p* = 0.004).

### Haplotype relationships and divergent times

**Fig. 5:**
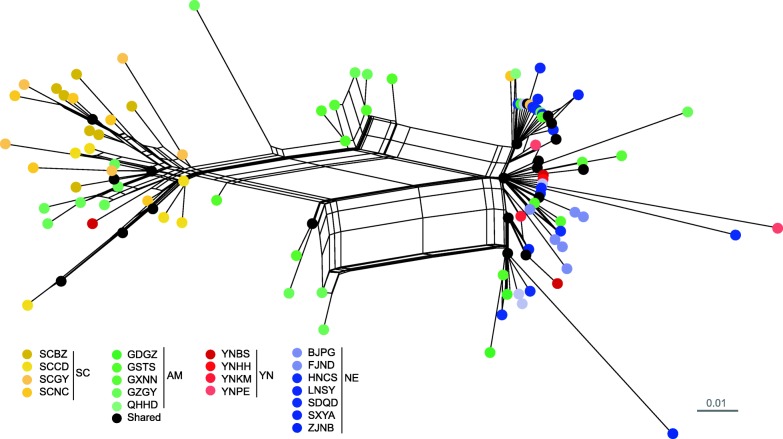
SPLITSTREE network for the *Grapholita molesta* haplotypes constructed by the method neighbor-net, based on combined mitochondrial genes. Circles of the same color indicate haplotypes from the same population. ‘Shared’ indicates the haplotype is shared by different populations. No unique haplotype was found in population YNQJ

Based on combined mitochondrial genes, the SPLITSTREE analysis revealed two major lineages and two intermediate lineages among haplotypes (Fig. 5). One major lineage was composed of haplotypes from Sichuan and its surrounding populations. The other one consisted of haplotypes mostly from Yunnan and the NE populations. The two intermediate lineages between the two major lineages were composed of individuals from populations located between or around Yunnan and Sichuan. The divergence time between major lineages of Yunnan and Sichuan OFM was estimated to be 329.2 (95% highest posterior density: 204.6–461.5) kya, while that between populations of Sichuan and populations with admixture was 227.3 (95% highest posterior density: 113.2–309.2) kya (Additional file 1: Figure S1). TSC analysis revealed similar topology of haplotype relationships to SPLITSTREE with three major groups of haplotypes. Haplotype 4, a widely distributed haplotype in 14 populations, was inferred as the ancestral haplotype (Additional file 1: Figure S2).

### Demographic history and gene flow

BEAST analysis showed relatively constant effective population sizes in each analyzed population (Additional file 1: Figure S3).

In DIYABC analysis, scenario 3 was supported with the highest posterior probability in 15 of the 32 data sets in step 1, with the posterior probabilities ranging from 0.1681 to 0.5064, which is an overwhelming advantage and far higher than a mean posterior probability, suggesting that NE populations were a mixture of Yunnan and Sichuan populations (Additional file 1: Table S2, Figure S4). Scenarios 8 and 9, in which Yunnan was derived from NE and was an admixture of NE and Sichuan, respectively, were alternatively supported in data sets when a relatively more admixed population of YNBS or SCGY was included (see Fig. 2 and Fig. 3). In step 2, 14 of 15 datasets supported the notion that the Sichuan populations had split from Yunnan, the posterior probabilities located between 0.3985 and 0.8630 (Additional file 1: Table S2). In step 3, the results showed that GXNN and GDGZ were an admixture from Yunnan and NE populations, while GSTS was an admixture of populations from Sichuan and NE, and QHHD had split from populations of NE (Additional file 1: Table S2).

IMa2 analysis showed that the migration rate from Yunnan to Sichuan populations was 0.97, while the reverse direction was 0.55, suggesting higher gene flow from Yunnan to Sichuan.

### Distribution of the two genetic clusters of OFM

**Fig. 6:**
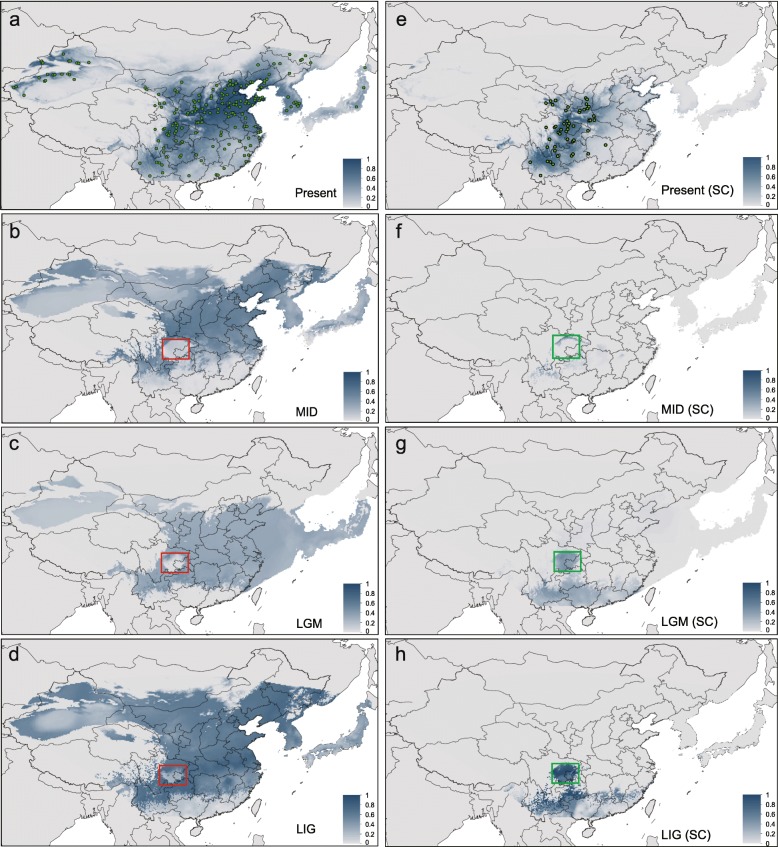
Potential distribution range of *Grapholita molesta* estimated by hindcasting the present niche model onto the last interglacial (LIG, 120 kya), LGM (21 kya) and mid-Holocene (MID, 6 kya) periods in East Asia using Maxent. **a** The current potential distribution using occurrences of total populations; (**b**-**d**) the inferred distribution range indicated from figure **a** onto the MID (**b**), LGM (**c**) and LIG (**d**) periods. **e** The current potential distribution using occurrences of Sichuan lineage; (**f**-**h**) the inferred distribution range hindcasted from figure **e** onto the MID (**f**), LGM (**g**) and LIG (**h**) periods. Green points represent the occurrences used in this study (**a**, **e**). Green and red boxes show the location of Sichuan Basin

When all occurrence sites were used in analysis, the average testing AUC (area under the curve) for replicate runs of current potential distribution was 0.859 and the standard deviation was 0.01. The modeled current potential distribution of OFM was basically the same to its actual distribution (Fig. 6a). OFM is a widespread species in the present and in the past times, when projecting the current niche into historical climate conditions (Fig. 6a-d). The potential distribution of OFM showed the most widely distribution at LIG period. The north populations shrunken during LGM when the ice age arrived. With the warming of temperature after LGM, populations expanded to north and south at MID and current conditions. Sichuan Basin and Yunnan is unfavorable for OFM at the past climatic conditions when all occurrence sites were included in analysis (red boxes in Fig. 6b-d).

When occurrence sites of Sichuan lineage were used in analysis, the present and past potential distributions of the OFM are shown in Fig. 6e-h. The average AUC values were 0.963, and the standard deviation was 0.007. The current potential distribution of Sichuan lineage shows a continuous range in the southwest of China, Sichuan basin, Qinling Mountains, and Loess Plateau (Fig. 6e). At the past climatic conditions, the distribution of Sichuan lineage was always complementary with that estimated by all occurrence sites (Fig. 6a-d). At the LIG, the distribution range was fragmented into several parts, and it was mainly in Yunnan-Guizhou plateau and Sichuan Basin which became less suitable during the LGM period (Fig. 6g-h). At the MID, the distribution range contracted greatly and was mainly located around Sichuan Basin (Fig. 6f). Sichuan lineage always distributed in or around Sichuan Basin and Yunnan during the Pleistocene climatic fluctuations. It seems that Sichuan Basin and Yunnan is not suitable for the majority OFM (red boxes in Fig. 6b-d) while suitable for Sichuan lineage (green boxes in Fig. 6f-h) at the past climate conditions.

We evaluated the niche overlap between Sichuan lineage (Fig. 2b) and the total OFM populations, in order to investigate whether the niche of the Sichuan lineage has undergone differentiation from other populations during the climatic fluctuations. PCA-env analyses showed that the niche in the Sichuan lineage had a moderated overlap (D = 0.465) with total populations (Additional file 1: Figure S5a-b). The niche equivalency is rejected, and the observed niche overlap is significantly smaller than simulated overlap (*P* < 0.01; Additional file 1: Figure S5d), indicating that the environmental niche of Sichuan lineage has differentiated from other populations. The niche similarity test of Sichuan lineage to total OFM populations is significant (*P* < 0.01; Additional file 1: Figure S5e), suggesting that niches of Sichuan lineage are more different to total OFM populations than expected by chance. However, niche similarity of total OFM populations to Sichuan lineage fell within the 95% confidence limits of the null distributions (*P* = 0.088; Additional file 1: Figure S5e), leading to non-rejection of the hypothesis that total OFM populations retained niche similarity to Sichuan lineage. These results were consistent with Maxent and molecular results that the distribution of Sichuan lineage was restricted in a relatively small region at current and past climate condition, while the other lineages expand to the whole current distribution range.

## Discussion

In this study, we investigated the population genetic structure and demographic history of OFM in its purported native range mainly around Yunnan and Sichuan. By dense sampling and using phylogeographical and ecological niche modelling approaches, we were able to identify putative glacial refugia of this species.

Both nuclear and mitochondrial markers unveiled strong genetic structure in populations of OFM, as previously found for this species in China [41]. Three distinct genetic groups were identified, corresponding to the Yunnan, Sichuan and NE groups, as well as a set of populations composed of multiple genetic clusters surrounding Yunnan and Sichuan groups. However, no group or population set showed a particularly high level of genetic diversity for either set of markers (Table 1).

Based on the identified population genetic structure, there are two possible scenarios around OFM refugia. One of these has refugia in locations with highly admixed populations. This pattern has been found in other species (Boyer, 2016, ME, mite; Carnaval 2009, Science), reflecting the fact that populations in refugia areas represent sinks to which other populations disperse. However, the three populations of OFM with the highest levels of admixed clusters are located in higher latitude and altitude regions compared to Yunnan and Sichuan, making them unlikely to represent sinks. Further analysis using DAPC revealed that these admixed populations are at intermediate positions between the Yunnan and Sichuan groups.

In species with a single refugium, a star-like haplotype network and decreased genetic diversity from the refugium to newly colonized areas are expected [46], neither of which was observed in OFM. The presence of admixed populations around Sichuan indicates that the Sichuan Basin served as a refugium separate from Yunnan. Multiple refugia were also supported in the IBD analyses, which indicated a lack of IBD generally but the presence of IBD when populations from Sichuan and the admixed region were excluded (for mitochondrial genes). After the dispersal of OFM from Yunnan to Sichuan, it is likely that a glacial event led to the separation of the two groups, while during interglacial periods intermediate areas were recolonized from adjacent regions as evidenced in our DIYABC analysis. Multiple refugia with this kind of pattern have been identified in many species in Europe and North America [6–8, 11].

We validated refugia by niche modelling the distribution of OFM under palaeoclimates. Species distribution models reconstruct past distribution of species by projecting the relationship between empirical observations and environmental data to earlier time periods using palaeoclimatic data [15, 47]. This approach can resolve complex evolutionary histories when integrating with molecular data [5, 16, 48]. Niche model analysis showed the distribution areas predicted for Sichuan lineage and total populations were highly complementary in Sichuan area. These results were consistent with those of IMa2 and DIYABC analysis, which gave powerful evidence that the Sichuan region survived as another refugium during the palaeoclimates and the Sichuan population is strongly an endemic one. Although the estimated time on the formation of the two refugia in Sichuan and Yunnan (ca. 329.2 kya) is earlier than the most ancient climate data that we can retrieve, our results indicated conditions in Yunnan and Sichuan were most likely suitable for the survival of OFM in the Quaternary. Niche modelling analysis using all occurrence sites showed Sichuan Basin and Yunnan was unfavorable for OFM but widespread in other regions at past climatic conditions. When we constructed niche model using Sichuan lineages, it showed Sichuan and Yunnan was suitable for this lineage of OFM, but other regions were unsuitable. It may indicate a niche shift of mixture populations compared to two original populations (Sichuan and Yunnan).

As far as we are known, this is the first identification of Yunnan and Sichuan Basin jointly as refugia in East Asia. Both regions located in the eastern Qinghai-Tibet Plateau, which is the highest and largest plateau in the world. Since QTP is known to have been affected by large climatic changes in the Quaternary [19], regions including Southeast China Hills, Yunnan and Sichuan Basin likely provide refugia during glaciation, although this remains controversial. The Yunnan region has a subtropical climate in southwestern China and represents the native range of OFM [41] as well as many other species [49–51]. Although Sichuan Basin has a higher latitude than Yunnan, the basin has a lower altitude resulting in warm temperatures during the glacial period compared to other areas at a similar latitude. This region has also been identified as a refugium of several species [29, 32, 50–53]. These studies either identified local-scale refugia in endemic species within one of these regions [50, 51], or multiple refugia widely distributed in eastern Asia as well as the adjacent regions of eastern QTP [28, 29, 32, 52]. Yunnan province located at the southeastern QTP and southern Yunnan-Guizhou Plateau (YGP), while the Sichuan Basin located at western of QTP and north of YGP. Both the QTP and YGP block the northern branch of the Indian monsoon circulation to northern and eastern China [54, 55]. Impacted by the QTP and YGP, the temperature is warmer in southern Yunnan (also low in altitude) and colder in northern Yunnan (also high in altitude). Decline in temperature during glaciation period may be the main factor that leads to the separation of OFM populations between Yunnan and Sichuan Basin by the high-altitude areas in northern Yunnan. Our study supports the boundary regions of eastern QTP as important refugia for species of this region.

Based on mitochondrial genes, we trace the divergence time of OFM between Yunnan and Sichuan to 329.2 kya, which is coincident with the first stage of Penultimate Glacial Maximum (PGM) (333–316 kya) [56] as estimated in Wei et al. (2015) [41]. The admixture events occurred at 227.3 kya, and also fall into the PGM between the second (277–266 kya) and third (154–136 kya) periods [56], suggesting the postglacial colonization of species in PGM in eastern Asia. Thus, populations of the OFM experienced the two glacial periods (PGM and LGM). Both the formation of the two refugia and the admixture events of the OFM occurred in the PGM but not significantly impacted in the LGM. Postglacial dispersal of the OFM occurred from Yunnan refuge to the east and then to the north of China. This situation was different from that in Europe and North America, where most species expanded their ranges later, after LGM.

Our study showed endemic populations of OFM in Sichuan Basin. This indicated the strong influence of climate oscillations on the adaptation of the OFM before PGM, as evidenced by the presence of endemic species in these areas [50, 51]. Niche model analysis indicates a wide range distribution of the OFM, while the potential distribution of Sichuan lineage is mainly around Sichuan Basin. Several haplotypes from other areas were also found in Sichuan and haplotypes of Sichuan population were clustered to lineage of other populations. This likely reflects recent dispersal through human-mediated activities. OFM has dispersed around the world within 100 years from East Asia to nearly all areas where its host plants occur [39, 40], pointing to a high capacity for dispersal. Genetic lineages restricted to the Sichuan Basin might have become locally adapted during glacial periods to a humid subtropical monsoonal climate while this remains to be tested.

## Conclusion

This study revealed strong genetic structure around the suspected native range of OFM. Two refugial regions of OFM were identified in Yunnan and Sichuan Basin, likely shaped in the first stage of the PGM. Analyses based on both microsatellites and mitochondrial genes showed haplotypes restricted to Sichuan, suggesting an endemic population in this region. The findings suggest a strong impact of the Quaternary climate on the genetic structure of OFM. Further study using genome-wide SNP could help to investigate the structure of populations in this region in more detail and reveal the adaptive mechanism of OFM to local climate oscillations in the Quaternary.

## Methods

### Sample collection and DNA extraction

Larvae of OFM were sampled from peach orchards on different trees to avoid the collection of siblings. In total 461 individuals were collected from 21 populations across China (Table 3, Fig. 2). All of them were preserved in absolute ethanol and stored at − 80 °C prior to DNA extraction. Total genomic DNA was extracted from one third to one-half of individual larvae using the DNeasy Blood and Tissue Kit (Qiagen, Germany), according to the manufacturer’s instructions.

**Table 3:** Collection information of the *Grapholita molesta* used in the study

Group	Population	Collection location	Longitude (°E)	Latitude (°N)	Number of individuals
YN	YNPE	Yunnan Province, Puer	101.04	22.75	17
YN	YNBS	Yunnan Province, Baoshan	99.17	25.15	24
YN	YNHH	Yunnan Province, Honghe	103.71	23.53	19
YN	YNQJ	Yunnan Province, Qujing	104.15	26.28	24
YN	YNKM	Yunnan Province, Kunming	102.67	25.16	24
SC	SCCD	Sichuan Province, Chengdu	104.07	30.67	24
SC	SCNC	Sichuan Province, Nanchong	105.98	31.16	24
SC	SCBZ	Sichuan Province, Bazhong	106.66	31.84	22
SC	SCGY	Sichuan Province, Guangyuan	105.89	32.64	22
AM	GDGZ	Guangdong Province, Guangzhou	113.50	22.99	24
AM	GXNN	Guangxi Province, Nanning	108.40	22.79	20
AM	GZGY	Guizhou Province, Guiyang	106.67	26.34	24
AM	GSTS	Gansu Province, Tianshui	105.72	34.58	23
AM	QHHD	Qinghai Province, Haidong	102.82	36.32	24
NE	HNCS	Hunan Province, Changsha	112.86	28.28	12
NE	FJND	Fujian Province, Ningde	119.55	26.67	23
NE	ZJNB	Zhejiang Province,Ningbo	121.54	29.87	18
NE	SXYA	Shanxi Province, Yanan	109.49	36.59	24
NE	SDQD	Shandong Province, Qingdao	120.43	36.16	21
NE	BJPG	Beijing City, Pinggu	117.12	40.14	24
NE	LNSY	Liaoning Province, Shenyang	123.43	41.81	24

YN, SC, NE indicates the Yunnan, Sichuan and north and east groups, while AM indicates the populations with the relatively high level of admixture. The populations underlined are those used in generating data sets for DIYABC analyses

### Data generation

For mitochondrial markers, two protein-coding genes (*cox1* and *nad5*) with a total length of 1386 bp were sequenced according to the method of [41] (Additional file 1: Table S3). Mitochondrial gene sequences were individually assembled using the software SEQMAN in the LASERGENE suite package version 7.1.2 (DNASTAR, Inc., USA). Each gene was aligned using CLUSTALW [57] implemented in MEGA version 7 [58] under default parameters. Alignment of the nucleotide sequences was checked and realigned following the guidance of amino acid alignment. For nuclear markers, 12 microsatellite loci developed from randomly sequenced genomic sequences were used [59] (Additional file 1: Table S3). The microsatellites were genotyped using GENEMAPPER version 4.0 (Applied Biosystems). The stuttering and large allele dropouts were detected using MICRO-CHECKER version 2.2.3 [60] and checked back in GENEMAPPER.

### Statistical power of microsatellite loci and genetic diversity analysis

To assess the statistical power affected by optional combination including the number of samples, sample sizes, number of loci and alleles as well as allele frequencies, POWSIM version 4.1 [61] was used, which is a simulation-based computer program that estimates power using chi-square and Fisher’s exact tests when evaluating the hypothesis of genetic homogeneity and degree of differentiation. A total of 2000 replicates were performed using population sizes and allele frequencies from the data, an effective population size of 4000 and drift generations varying from 8 to 80 to obtain expected *F*_*ST*_ values from 0.001 to 0.01.

For microsatellite loci, allele frequencies, observed heterozygosity (*H*_*O*_), expected heterozygosity (*H*_*E*_) and the polymorphic information content (PIC) were analyzed using the macros in Microsatellite Tools [62]. Deviation from Hardy–Weinberg equilibrium (HWE) and the linkage disequilibrium (LD) were tested by GENEPOP version 4.0.11 [63]. We estimated null allele frequencies using the software FREENA [64]. The allelic richness (*A*_*R*_) of each locus and inbreeding coefficient (*F*_*IS*_) between individuals within each geographical population were detected using FSTAT version 2.9.3 [65].

For mitochondrial genes, polymorphic sites, DNA polymorphism, linkage disequilibrium, the number of polymorphic sites (*S*), total number of mutations (*η*), number of haplotypes (*H*), haplotype diversity (*Hd*), nucleotide diversity (*Pi*), Tajima’s D and average number of nucleotide differences (*K*) from both single and combined mitochondrial sequences were calculated in DnaSP version 5.10.01 [66].

### Population genetic structure analysis

Considering the advantages of various of software and methodologies, we applied multiple complementary approaches, including POPTREE2, BAPS, STRUCTURE, DAPC and isolation-by-distance (IBD) analyses, to explore the population genetic structure of OFM.

First, to reveal the phylogenetic relationships among populations, POPTREE2 [67] was used based on both microsatellites and/or mitochondrial genes using the neighbor-joining method under D_*A*_ distance. POPTREE2 constructs phylogenetic trees from allele frequency data by using the neighbor-joining (NJ) method and the unweighted pair-group method with arithmetic mean (UPGMA) [67].

Second, a Bayesian analysis of population genetic structure (BAPS) analysis was conducted using BAPS version 6.0 [68] based on microsatellites and mitochondrial genes. The spatially explicit BAPS model treats both the allele frequencies of the molecular markers and the number of genetically diverged groups in populations as random variables, as well as combining sample locations with genetic data, especially with large data sets. We performed 30 repeat runs, of which the maximum number of genetically diverged groups for the mitochondrial gene (*K*) was set to 5, 10, 15 and 20 respectively to ensure convergence and consistency of the results because the initial *K* value may affect the initial assignment of simulation and reduce the possibility of finding only a local mode.

Third, the population genetic structure was analyzed by STRUCTURE version 2.3.4 [69] based on microsatellites. This method can identify subsets of the whole sample by detecting allele frequency differences within the data and can assign individuals to those sub-populations based on analysis of likelihoods. We used 30 replicates with *K* from 1 to 10, and a burn-in of 500,000 iterations followed by 2000,000 Markov Chain Monte Carlo iterations to identify the number of clusters. All results were collected and submitted to the online software STRUCTURE HARVESTER version 0.6.94 [70] to determine the optimal *K* using both the Ln Pr (X|*K*) and the Delta (*K*) methods.

Fourth, discriminant analysis of principal components (DAPC) implemented in the R package ADEGENET 1.4–2 [71] was used to reveal population structure based on microsatellites. This method does not require a biological hypothesis and acts as a complement to STRUCTURE in that it considers HWE and linkage equilibrium in its own algorithm.

Finally, IBD was tested in populations of OFM using a Mantel test correlating genetic distance (*F*_*ST*_/(1-*F*_*ST*_)) and geographic distance (ln km) using *ade4* version 1.7–4 implemented in R (Daniel et al. 2004) with 999 replicates. This method was also used to test the correlation between *F*_*ST*_ values calculated from microsatellites and mitochondrial genes. The values of *F*_*ST*_ were calculated in FREENA version 4.0 with ENA (excluding null alleles) model [72] for microsatellite data and ARLEQUIN suit version 3.5 for mitochondrial genes [73].

### Haplotype relationship analysis

We used SPLITSTREE version 4.14.5 [74] to explore relationships among mitochondrial haplotypes. The program takes as input a set of taxa represented by aligned sequences and produces output trees or networks using different methods and provides a framework for evolutionary analysis using both trees and networks. The network methods combine reticulation events and phylogenetic topology into a visualized interface. We performed a Neighbor-Net analysis for construction of the network with a Kimura-2-parameter distance model [75].

Taking advantage of the mitochondrial molecular clock estimated in insects [76], we estimated the divergence time between the major lineages of mitochondrial haplotypes by BEAST version 1.8.1 [77] as described in Wei et al. [41]. The sibling species *Grapholita dimorpha* was used as an outgroup (GenBank Number: KJ671625) [78].

We also constructed a haplotype network based on statistical parsimony in the program TCS version 1.21 [79] to reveal the haplotype relationship and to identify the ancestral haplotype.

### Demographic history analysis

First, we investigated the population history by estimating the changes in the effective population size over time using a Bayesian skyline plot (BSP) [80] implemented in the software BEAST version 1.8.0 [77]. The piecewise-linear skyline model was selected for the Bayesian skyline coalescent tree priors. Chains were run for 200 million generations with a sampling of every 20,000 generations.

Second, we used an ABC method implemented in DIYABC version 2.1.0 [81] to infer the demographic history of OFM, using the microsatellite data. In this method, different scenarios were proposed and compared based on prior knowledge. We used different data sets to test the scenarios as suggested in Lombaert, E, et al. [82]. Based on the results of population genetic structure analyses, the 21 populations were classified into three major groups and a set of populations with a high level of admixture. For each major group, we chose populations with the least admixture and most admixture as representatives (Table 3). These populations were assembled into 32 data sets with one representative from each group (Additional file 1: Table S2). To reduce the complexity of the tested scenarios, we used a hierarchical approach [6] by optimizing the procedure into three successive steps.

In step 1, the relationships among the three major groups were compared by using the 32 assembled data sets. A total of nine possible scenarios were tested, regarding split and admixture events.

In step 2, two competing scenarios regarding the ancestral population were tested based on the results of step 1, of which one population was derived from the admixture of the other two populations. Fifteen datasets that supported the most plausible scenario in step 1 were retained for analysis in this step.

In step 3, the origins of four populations with admixture, i.e., GXNN, GDGZ, GSTS, QHHD, were tested individually by adding them to the best scenario of step 2. A hierarchical tournament approach was used in this step [6], in which two subsets of scenarios (step 3–1 refers to the northern and eastern group [NE] while step 3–2 does not include NE with 3 scenarios in each step and 56 and 32 datasets used, respectively) are compared in the first round of analyses and the winners are compared by posterior probabilities.

Methods for scenario comparisons and validation are described in [41]. Based on results of Bayesian skyline plot analysis, constant effective population size was assumed in all scenarios. Scenarios and priors used in DIYABC analysis are shown in supplemental information (Additional file 1: Table S2, Table S4, Figure S6-S9).

Third, gene flow between Sichuan and Yunnan populations was estimated using the isolation-by-migration (IM) model in IMa2 [83, 84] based on microsatellites. The program implements a method for generating posterior probabilities for complex demographic population genetic models. For the M mode, a 6-h burn-in was applied. The number of steps between genealogy saving was defaulted as 100 and the outputs were exported every 6 h. The calculation model was fixed to include ranges on mutation rates as priors on mutation rate scalars. A geometric heating mode was used with 5 chains. The first and second heating parameters were set to 0.999 and 0.3, respectively. The prior value of migration was defined as 5 and the maximum population size and maximum time of population splitting were 150 and 50, respectively. The migration prior follows an exponential distribution with mean given by -m or a parameter prior file was used. Multiple run options together with the output options were used to improve the comprehensiveness and completeness of the results. Several runs were carried out with different random seeds to generate 100,000 genealogies for L mode analysis. All genealogy outputs calculated by the M mode were collected for the L mode and the final result was visualized by the program IMfig [85].

### Niche model analysis

For niche modelling, 326 occurrences, throughout its entire distribution range in East Asia, were obtained from the published literature, websites (CABI) and collected samples in our laboratory. Occurrences were rarefied to 178 sites by excluding imprecise points and retaining one occurrence in a grid cell (30 min of a longitude/latitude degree). Seven bioclimatic variables [86] that most likely restrict the distribution of OFM [87–89] were acquired from the WorldClim website at a resolution of 10-min, including annual mean temperature (BIO1), mean diurnal temperature range (BIO2), maximum temperature of the warmest month (BIO5), minimum temperature of the coldest month (BIO6), annual mean precipitation (BIO12), precipitation of wettest month (BIO13) and precipitation of driest month (BIO14). The maximum entropy implemented in MAXENT version 3.4.1 [15] was used to estimate the possible distribution of OFM in the present and past times. Models was constructed using bootstrap with 10 replicates. The area under the curve (AUC) of a receiver operating characteristic plot was used to evaluate model performance. The potential distribution during the last interglacial (LIG, 120 kya), LGM (21 kya) and mid-Holocene (MID, 6 kya) was estimated by projecting the current niche model onto climatic conditions during the LIG, LGM and MID periods. The past climatic conditions were download from the WorldClim website which were downscaled and calibrated from current bioclimatic variables using the Community Climate System Model 3 (CCSM 3) [90].

Both niche modelling and molecular data revealed that populations around Sichuan Basin were distinct from other regions (see results section). We obtained 49 occurrence sites located in the distribution areas of Sichuan lineage (dash line circle in Fig. 2b, including Sichuan and Yunnan areas) for modelling. The current and past distribution of the two lineages was estimated as described above. Niche overlaps between Sichuan lineage and all lineages were measured using principal component analysis on the environment spaces (PCA-env) in the R script following Broennimann et al. [91]. PCA-env analyses compute density of occurrences in the environmental space and compare niche overlap using Schoener’s D metric [92]. The statistical tests of niche equivalency and similarity were conducted with 500 iterations by comparing the observed D values to simulated overlap distribution. The niche equivalency examines whether the niche overlap is constant by randomly reallocating the occurrences of both lineages among the two ranges, while the niche similarity test evaluates whether the niche occupied by one lineages is more similar to the one occupied by the other lineage than expected by chance [91].

## Additional file

Additional file 1: **Table S1.** Results of POWSIM analysis. **Table S2.** The best scenario estimated in each step by using each data set. **Table S3.** Primers used in the study. **Table S4.** Priors used in DIYABC analysis. **Figure S1.** Dating tree of divergence time for each *Grapholita molesta* group. **Figure S2.** Statistical parsimony networks of the combined mitochondrial genes. **Figure S3.** Bayesian sky plot analysis on the variation in effective population size using BEAST in 12 populations. **Figure S4.** Representative illustration on the pre-evaluation, model checking, posterior distribution and posterior probability in DIYABC analyses. **Figure S5.** Comparison of the distribution range of the clusters from Sichuan (SC) and clusters from other regions. **Figure S6.** Scenarios in Step 1 of DIYABC analysis. **Figure S7.** Scenarios in Step 2 of DIYABC analysis. **Figure S8.** Scenarios in Step 3–1 of DIYABC analysis. **Figure S9.** Scenarios in Step 3–2 of DIYABC analysis. (PDF 2416 kb)

## Abbreviations

ABC: Approximate Bayesian Computation
AUC: Area under the curve
BAPS: Bayesian analysis of population genetic structure
bp: Base pairs
BSP: Bayesian skyline plot
CCSM 3: Community Climate System Model 3
DAPC: Discriminant analysis of principal components
ENA: Excluding null alleles
HWE: Hardy–Weinberg equilibrium
IBD: Isolation by distance
IM: Isolation-by-migration
LD: Linkage disequilibrium
LGM: Last Glacial Maximum
LIG: The last interglacial
MID: Mid-Holocene
NE: The northern and eastern group
NJ: Neighbor-joining
OFM: Oriental fruit moth
PCA-env: Principal component analysis on the environment spaces
PGM: Penultimate Glacial Maximum
PIC: Polymorphic information content
QTP: Qinghai-Tibetan Plateau
UPGMA: Unweighted pair-group method with arithmetic mean
YGP: Yunnan-Guizhou Plateau

## References

[1] Hewitt G (2000). The genetic legacy of the quaternary ice ages. Nature.

[2] Hewitt G (2004). Genetic consequences of climatic oscillations in the quaternary. Philos Trans R Soc Lond B Biol Sci.

[3] Svenning J-C, Eiserhardt WL, Normand S, Ordonez A, Sandel B (2015). The influence of paleoclimate on present-day patterns in biodiversity and ecosystems. Annu Rev Ecol Evol Syst.

[4] Hewitt GM (2001). Speciation, hybrid zones and phylogeography—or seeing genes in space and time. Mol Ecol.

[5] Bidegaray-Batista L, Sanchez-Gracia A, Santulli G, Maiorano L, Guisan A, Vogler AP, Arnedo MA (2016). Imprints of multiple glacial refugia in the Pyrenees revealed by phylogeography and palaeodistribution modelling of an endemic spider. Mol Ecol.

[6] Stone GN, White SC, Csóka G, Melika G, Mutun S, Pénzes Z, Sadeghi SE, Schönrogge K, Tavakoli M, Nicholls JA (2017). Tournament ABC analysis of the western Palaearctic population history of an oak gall wasp, Synergus umbraculus. Mol Ecol.

[7] Kolar F, Fuxova G, Zaveska E, Nagano AJ, Hyklova L, Lucanova M, Kudoh H, Marhold K (2016). Northern glacial refugia and altitudinal niche divergence shape genome-wide differentiation in the emerging plant model *Arabidopsis arenosa*. Mol Ecol.

[8] Sim Z, Hall JC, Jex B, Hegel TM, Coltman DW (2016). Genome-wide set of SNPs reveals evidence for two glacial refugia and admixture from postglacial recolonization in an alpine ungulate. Mol Ecol.

[9] Godefroid M, Rocha S, Santos H, Paiva MR, Burban C, Kerdelhué C, Branco M, Rasplus JY, Rossi JP (2016). Climate constrains range expansion of an allochronic population of the pine processionary moth. Divers Distrib.

[10] Alberdi A, Gilbert MTP, Razgour O, Aizpurua O, Aihartza J, Garin I (2015). Contrasting population-level responses to Pleistocene climatic oscillations in an alpine bat revealed by complete mitochondrial genomes and evolutionary history inference. J Biogeogr.

[11] Comps B, Gömöry D, Letouzey J, Thiébaut B, Petit R (2001). Diverging trends between heterozygosity and allelic richness during postglacial colonization in the European beech. Genetics.

[12] Stone GN, Challis RJ, Atkinson RJ, CSÓKA G, Hayward A, Melika G, Mutun S, Preuss S, Rokas A, Sadeghi E (2007). The phylogeographical clade trade: tracing the impact of human-mediated dispersal on the colonization of northern Europe by the oak gallwasp Andricus kollari. Mol Ecol.

[13] Gillespie RG, Baldwin BG, Waters JM, Fraser CI, Nikula R, Roderick GK (2012). Long-distance dispersal: a framework for hypothesis testing. Trends Ecol Evol.

[14] Pelletier TA, Carstens BC (2014). Model choice for phylogeographic inference using a large set of models. Mol Ecol.

[15] Phillips SJ, Anderson RP, Schapire RE (2006). Maximum entropy modeling of species geographic distributions. Ecol Model.

[16] Schorr G, Pearman PB, Guisan A, Kadereit JW (2013). Combining palaeodistribution modelling and phylogeographical approaches for identifying glacial refugia in alpine primula. J Biogeogr.

[17] Davis MB, Shaw RG (2001). Range shifts and adaptive responses to quaternary climate change. Science.

[18] Inoue Kentaro, Lang Brian K., Berg David J. (2015). Past climate change drives current genetic structure of an endangered freshwater mussel species. Molecular Ecology.

[19] Shi YF, Zhao JD, Wang J (2011). New understanding of quaternary glaciations in China: Shanghai popular science press.

[20] Tian S, Kou Y, Zhang Z, Yuan L, Li D, Lopez-Pujol J, Fan D, Zhang Z (2018). Phylogeography of Eomecon chionantha in subtropical China: the dual roles of the Nanling Mountains as a glacial refugium and a dispersal corridor. BMC Evol Biol.

[21] Zeng YF, Wang WT, Liao WJ, Wang HF, Zhang DY (2015). Multiple glacial refugia for cool-temperate deciduous trees in northern East Asia: the Mongolian oak as a case study. Mol Ecol.

[22] Li YC, Zhong DL, Rao GY, Wen J, Ren Y, Zhang JQ (2018). Gone with the trees: Phylogeography of Rhodiola sect. Trifida (Crassulaceae) reveals multiple refugia on the Qinghai-Tibetan plateau. Mol Phylogenet Evol.

[23] Meng L, Yang R, Abbott RJ, Miehe G, Hu T, Liu J (2007). Mitochondrial and chloroplast phylogeography of Picea crassifolia Kom. (Pinaceae) in the Qinghai-Tibetan plateau and adjacent highlands. Mol Ecol.

[24] Myers N, Mittermeier RA, Mittermeier CG, Da Fonseca GA, Kent J (2000). Biodiversity hotspots for conservation priorities. Nature.

[25] Opgenoorth L, Vendramin GG, Mao K, Miehe G, Miehe S, Liepelt S, Liu J, Ziegenhagen B (2010). Tree endurance on the Tibetan plateau marks the world’s highest known tree line of the last glacial maximum. New Phytol.

[26] Wang H, Qiong L, Sun K, Lu F, Wang Y, Song Z, Wu Q, Chen J, Zhang W (2010). Phylogeographic structure of Hippophae tibetana (Elaeagnaceae) highlights the highest microrefugia and the rapid uplift of the Qinghai-Tibetan plateau. Mol Ecol.

[27] Bai WN, Liao WJ, Zhang DY (2010). Nuclear and chloroplast DNA phylogeography reveal two refuge areas with asymmetrical gene flow in a temperate walnut tree from East Asia. New Phytol.

[28] Zhao C, Wang CB, Ma XG, Liang QL, He XJ (2013). Phylogeographic analysis of a temperate-deciduous forest restricted plant (*Bupleurum longiradiatum* Turcz.) reveals two refuge areas in China with subsequent refugial isolation promoting speciation. Mol Phylogenet Evol.

[29] Ying LX, Zhang TT, Chiu CA, Chen TY, Luo SJ, Chen XY, Shen ZH (2016). The phylogeography of *Fagus hayatae* (Fagaceae): genetic isolation among populations. Ecol Evol.

[30] Zhao XL, Gao XF, Zhu ZM, Gao YD, Xu B (2017). The demographic response of a deciduous shrub (the Indigofera bungeana complex, Fabaceae) to the Pleistocene climate changes in East Asia. Sci Rep.

[31] Bai WN, Wang WT, Zhang DY (2016). Phylogeographic breaks within Asian butternuts indicate the existence of a phytogeographic divide in East Asia. New Phytol.

[32] Lu G, Lin A, Luo J, Blondel DV, Meiklejohn KA, Sun K, Feng J (2013). Phylogeography of the Rickett's big-footed bat, *Myotis pilosus* (Chiroptera: Vespertilionidae): a novel pattern of genetic structure of bats in China. BMC Evol Biol.

[33] Ding L, Gan XN, He SP, Zhao EM (2011). A phylogeographic, demographic and historical analysis of the short-tailed pit viper (*Gloydius brevicaudus*): evidence for early divergence and late expansion during the Pleistocene. Mol Ecol.

[34] Li SH, Yeung CKL, Feinstein J, Han LX, Manh HL, Wang CX, Ding P (2009). Sailing through the Late Pleistocene: unusual historical demography of an east Asian endemic, the Chinese Hwamei (*Leucodioptron canorum canorum*), during the last glacial period. Mol Ecol.

[35] Meng XF, Shi M, Chen XX (2008). Population genetic structure of *Chilo suppressalis* (Walker)(Lepidoptera: Crambidae): strong subdivision in China inferred from microsatellite markers and mtDNA gene sequences. Mol Ecol.

[36] Tian S, Lei SQ, Hu W, Deng LL, Li B, Meng QL, Soltis DE, Soltis PS, Fan DM, Zhang ZY (2015). Repeated range expansions and inter−/postglacial recolonization routes of Sargentodoxa cuneata (Oliv.) Rehd. Et Wils. (Lardizabalaceae) in subtropical China revealed by chloroplast phylogeography. Mol Phylogenet Evol.

[37] Sakaguchi S, Qiu YX, Liu YH, Qi XS, Kim SH, Han J, Takeuchi Y, Worth JR, Yamasaki M, Sakurai S (2012). Climate oscillation during the quaternary associated with landscape heterogeneity promoted allopatric lineage divergence of a temperate tree Kalopanax septemlobus (Araliaceae) in East Asia. Mol Ecol.

[38] Qu Y, Zhang R, Quan Q, Song G, Li SH, Lei F (2012). Incomplete lineage sorting or secondary admixture: disentangling historical divergence from recent gene flow in the vinous-throated parrotbill (*Paradoxornis webbianus*). Mol Ecol.

[39] Rothschild GHL, Vickers RA, van der Geest L, Evenhuis H (1991). Biology, ecology and control of the oriental fruit moth. World Crop Pests Tortricid pests: their biology, natural enemies and control.

[40] Kirk H, Dorn S, Mazzi D (2013). Worldwide population genetic structure of the oriental fruit moth (*Grapholita molesta*), a globally invasive pest. BMC Ecol.

[41] Wei SJ, Cao LJ, Gong YJ, Shi BC, Wang S, Zhang F, Guo XJ, Wang YM, Chen XX (2015). Population genetic structure and approximate Bayesian computation analyses reveal the southern origin and northward dispersal of the oriental fruit moth *Grapholita molesta* (Lepidoptera: Tortricidae) in its native range. Mol Ecol.

[42] Silva-Brandão KL, Brandão MM, Omoto C, Sperling FA (2015). Genotyping-by-sequencing approach indicates geographic distance as the main factor affecting genetic structure and gene flow in Brazilian populations of *Grapholita molesta* (Lepidoptera, Tortricidae). Evol Appl.

[43] Timm AE, Geertsema H, Warnich L (2008). Population genetic structure of *Grapholita molesta* (Lepidoptera : Tortricidae) in South Africa. Ann Entomol Soc Am.

[44] Torriani MV, Mazzi D, Hein S, Dorn S (2010). Structured populations of the oriental fruit moth in an agricultural ecosystem. Mol Ecol.

[45] Zheng Y, Peng X, Liu G, Pan H, Dorn S, Chen M (2013). High genetic diversity and structured populations of the oriental fruit moth in its range of origin. PLoS One.

[46] Provan J, Bennett K (2008). Phylogeographic insights into cryptic glacial refugia. Trends Ecol Evol.

[47] Guisan A, Zimmermann NE (2000). Predictive habitat distribution models in ecology. Ecol Model.

[48] Komaki S, Igawa T, Lin SM, Tojo K, Min MS, Sumida M (2015). Robust molecular phylogeny and palaeodistribution modelling resolve a complex evolutionary history: glacial cycling drove recurrent mtDNA introgression among *Pelophylax* frogs in East Asia. J Biogeogr.

[49] Yang FS, Li YF, Ding X, Wang XQ (2008). Extensive population expansion of Pedicularis longiflora (Orobanchaceae) on the Qinghai-Tibetan plateau and its correlation with the quaternary climate change. Mol Ecol.

[50] Fan Z, Liu S, Liu Y, Liao L, Zhang X, Yue B (2012). Phylogeography of the South China field mouse (Apodemus draco) on the southeastern Tibetan plateau reveals high genetic diversity and glacial refugia. PLoS One.

[51] Yang Y, Ren L, Wang T, Xu L, Zong S. Comparative morphology of sensilla on antenna, maxillary palp and labial palp of larvae of Eucryptorrhynchus scrobiculatus (Olivier) and E.brandti (Harold) (Coleoptera: Curculionidae). Acta Zool. 2017;47(1):3–10.

[52] Flanders J, Wei L, Rossiter SJ, Zhang S (2011). Identifying the effects of the Pleistocene on the greater horseshoe bat, *Rhinolophus ferrumequinum*, in East Asia using ecological niche modelling and phylogenetic analyses. J Biogeogr.

[53] You Y, Sun K, Xu L, Wang L, Jiang T, Liu S, Lu G, Berquist SW, Feng J (2010). Pleistocene glacial cycle effects on the phylogeography of the Chinese endemic bat species, *Myotis davidii*. BMC Evol Biol.

[54] Shi Z, Sha Y, Liu X (2017). Effect of Yunnan–Guizhou topography at the southeastern Tibetan plateau on the Indian monsoon. J Clim.

[55] Zhisheng A, Guoxiong W, Jianping L, Youbin S, Yimin L, Weijian Z, Yanjun C, Anmin D, Li L, Jiangyu M (2015). Global monsoon dynamics and climate change. Annu Rev Earth Planet Sci.

[56] Yi C-L, Cui Z-J, Xiong H-G (2005). Numerical periods of quaternary glaciations in China. Quat Sci.

[57] Thompson JD (1994). CLUSTAL W:improving the sensitivity of progressive multiple sequence alignment through sequence weighting, positions-specific gap penalties and weight matrix choice. Nucleic Acids Res.

[58] Kumar S, Stecher G, Tamura K (2016). MEGA7: molecular evolutionary genetics analysis version 7.0 for bigger datasets. Mol Biol Evol.

[59] Song W, Cao LJ, Wang YZ, Li BY, Wei SJ (2017). Novel microsatellite markers for the oriental fruit moth *Grapholita molesta* (Lepidoptera: Tortricidae) and effects of null alleles on population genetics analyses. Bull Entomol Res.

[60] van Oosterhout C, Hutchinson WF, WILLS DPM, Shipley P (2004). Micro-checker: software for identifying and correcting genotyping errors in microsatellite data. Mol Ecol Notes.

[61] Ryman N, Palm S (2006). POWSIM: a computer program for assessing statistical power when testing for genetic differentiation. Mol Ecol Notes.

[62] Stephen P. Trypanotolerance in west african cattle and the population genetic effects of selection. Dublin: PhD. Thesis university of Dublin; 2001.

[63] Raymond M, Rousset F (1995). GENEPOP (version 1.2): population genetics software for exact tests and Ecumenicism. J Hered.

[64] Chapuis M-P, Estoup A (2007). Microsatellite null alleles and estimation of population differentiation. Mol Biol Evol.

[65] Goudet J (1995). FSTAT (version 1.2): a computer program to calculate F-statistics. J Hered.

[66] Librado P, Rozas J (2009). DnaSP v5: a software for comprehensive analysis of DNA polymorphism data. Bioinformatics.

[67] Takezaki N, Nei M, Tamura K (2010). POPTREE2: software for constructing population trees from allele frequency data and computing other population statistics with windows interface. Mol Biol Evol.

[68] Cheng L, Connor TR, Sirén J, Aanensen DM, Corander J (2013). Hierarchical and spatially explicit clustering of DNA sequences with BAPS software. Mol Biol Evol.

[69] Pritchard JK, Stephens M, Donnelly P (2000). Inference of population structure using multilocus genotype data. Genetics.

[70] Earl DA, vonHoldt BM (2011). STRUCTURE HARVESTER: a website and program for visualizing STRUCTURE output and implementing the Evanno method. Conserv Genet Resour.

[71] Jombart T, Devillard S, Dufour AB, Pontier D (2008). Revealing cryptic spatial patterns in genetic variability by a new multivariate method. Heredity.

[72] Rousset F (2008). Genepop ‘007: a complete re-implementation of the genepop software for windows and Linux. Mol Ecol Resour.

[73] Excoffier L, Lischer HEL (2010). Arlequin suite ver 3.5: a new series of programs to perform population genetics analyses under Linux and windows. Mol Ecol Resour.

[74] Huson DH, Bryant D (2006). Application of phylogenetic networks in evolutionary studies. Mol Biol Evol.

[75] Kimura M (1980). A simple method for estimating evolutionary rates of base substitutions through comparative studies of nucleotide sequences. J Mol Evol.

[76] Papadopoulou A, Anastasiou I, Vogler AP (2010). Revisiting the insect mitochondrial molecular clock: the mid-Aegean trench calibration. Mol Biol Evol.

[77] Drummond AJ, Rambaut A (2007). BEAST: Bayesian evolutionary analysis by sampling trees. BMC Evol Biol.

[78] Niu FF, Fan XL, Wei SJ (2016). Complete mitochondrial genome of the *Grapholita dimorpha* Komai (Lepidoptera: Tortricidae). Mitochondrial DNA A DNA Mapp Seq Anal.

[79] Clement M, Posada D, Crandall KA (2000). TCS: a computer program to estimate gene genealogies. Mol Ecol.

[80] Drummond A, Rambaut A, Shapiro B, Pybus O (2005). Bayesian coalescent inference of past population dynamics from molecular sequences. Mol Biol Evol.

[81] Cornuet J-M, Pudlo P, Veyssier J, Dehne-Garcia A, Gautier M, Leblois R, Marin J-M, Estoup A (2014). DIYABC v2.0: a software to make approximate Bayesian computation inferences about population history using single nucleotide polymorphism, DNA sequence and microsatellite data. Bioinformatics.

[82] Lombaert E, Guillemaud T, Lundgren J, Koch R, Facon B, Grez A, Loomans A, Malausa T, Nedved O, Rhule E (2014). Complementarity of statistical treatments to reconstruct worldwide routes of invasion: the case of the Asian ladybird *Harmonia axyridis*. Mol Ecol.

[83] Hey J, Chung Y, Sethuraman A (2015). On the occurrence of false positives in tests of migration under an isolation-with-migration model. Mol Ecol.

[84] Hey J (2010). The divergence of chimpanzee species and subspecies as revealed in multipopulation isolation-with-migration analyses. Mol Biol Evol.

[85] Shang HY, Li ZH, Dong M, Adams RP, Miehe G, Opgenoorth L, Mao KS (2015). Evolutionary origin and demographic history of an ancient conifer (Juniperus microsperma) in the Qinghai-Tibetan plateau. Sci Rep.

[86] Hijmans RJ, Cameron SE, Parra JL, Jones PG, Jarvis A (2005). Very high resolution interpolated climate surfaces for global land areas. Int J Climatol.

[87] Hansen JD (2002). Effect of cold temperature treatments on the mortality of eggs and feeding larvae of the oriental fruit moth. Horttechnology.

[88] Roberts WP, Proctor JR, Phillips JHH (1978). Effect of constant temperatures on number of larval instars of oriental fruit moth, Grapholitha-molesta (Lepidoptera-Tortricidae). Can Entomol.

[89] Chaudhry G-U (1951). The development and fecundity of the oriental fruit moth, *Grapholitha* ( *Cydia*) *molesta* (Busck) under controlled temperatures and humidities. Bull Entomol Res.

[90] Collins WD, Bitz CM, Blackmon ML, Bonan GB, Bretherton CS, Carton JA, Chang P, Doney SC, Hack JJ, Henderson TB (2006). The community climate system model version 3 (CCSM3). J Clim.

[91] Broennimann O, Fitzpatrick MC, Pearman PB, Petitpierre B, Pellissier L, Yoccoz NG, Thuiller W, Fortin MJ, Randin C, Zimmermann NE (2012). Measuring ecological niche overlap from occurrence and spatial environmental data. Glob Ecol Biogeogr.

[92] Schoener TW (1970). Nonsynchronous spatial overlap of lizards in patchy habitats. Ecology.

