# Supplementary material for: Multiple refugia from penultimate glaciations in East Asia demonstrated by phylogeography and ecological modelling of an insect pest
Source: BMC Evol Biol. 2018 Oct 11;18:152. doi: 10.1186/s12862-018-1269-z (PMC6186135; doi:10.1186/s12862-018-1269-z)
Supplement: Supplementary file 1 — Table S1. Results of POWSIM analysis. Table S2. The best scenario estimated in each step by using each data set. Table S3. Primers used in the study. Table S4. Priors used in DIYABC analysis. Figure S1. Dating tree of divergence time for each Grapholita molesta group. Figure S2. Statistical parsimony networks of the combined mitochondrial genes. Figure S3. Bayesian sky plot analysis on the variation in effective population size using BEAST in 12 populations. Figure S4. Representative illustration on the pre-evaluation, model checking, posterior distribution and posterior probability in DIYABC analyses. Figure S5. Comparison of the distribution range of the clusters from Sichuan (SC) and clusters from other regions. Figure S6. Scenarios in Step 1 of DIYABC analysis. Figure S7. Scenarios in Step 2 of DIYABC analysis. Figure S8. Scenarios in Step 3–1 of DIYABC analysis. Figure S9. Scenarios in Step 3–2 of DIYABC analysis. (PDF 2416 kb) [file 12862_2018_1269_MOESM1_ESM.pdf]

Table S1 Results of POWSIM analysis

Ne	t	F_{ST}	Chi2	Fisher
4000	8	0.0010	0.403	0.358
4000	20	0.0025	0.960	0.883
4000	40	0.0050	1.000	1.000
4000	80	0.0100	1.000	1.000

Ne: effective population size; t: generations; Chi2: statistical testing results based on Pearson's traditional contingency chi-square; Fisher: statistical testing results based on Fisher's exact test for general rxc tables.

Table S2 The best scenario estimated in each step by using each data set

Step	Dataset	Best scenario	Posterior probabilities [95% confidence interval]
Step 1	data01_SCCD_YNQJ_BJPG	scenario 3	0.2573 [0.2453,0.2694]
	data02_SCCD_YNQJ_LNSY	scenario 3	0.2459 [0.2336,0.2582]
	data03_SCCD_YNQJ_SXYA	scenario 3	0.2366 [0.2248,0.2484]
	data04_SCCD_YNQJ_FJND	scenario 3	0.3038 [0.2952,0.3123]
	data05_SCCD_YNHH_BJPG	scenario 3	0.5444 [0.5236,0.5653]
	data06_SCCD_YNHH_LNSY	scenario 3	0.4444 [0.4271,0.4617]
	data07_SCCD_YNHH_SXYA	scenario 3	0.4431 [0.4253,0.4608]
	data08_SCCD_YNHH_FJND	scenario 3	0.5548 [0.5135,0.5960]
	data09_SCCD_YNBS_BJPG	scenario 8	0.3932 [0.3772,0.4092]
	data10_SCCD_YNBS_LNSY	scenario 8	0.3191 [0.3079,0.3302]
	data11_SCCD_YNBS_SXYA	scenario 1	0.3463 [0.3358,0.3569]
	data12_SCCD_YNBS_FJND	scenario 8	0.3310 [0.3147,0.3473]
	data13_SCCD_YNPE_BJPG	scenario 3	0.2887 [0.2770,0.3003]
	data14_SCCD_YNPE_LNSY	scenario 3	0.3070 [0.2951,0.3190]
	data15_SCCD_YNPE_SXYA	scenario 8	0.2718 [0.2628,0.2809]
	data16_SCCD_YNPE_FJND	scenario 3	0.2909 [0.2809,0.3010]
	data17_SCGY_YNQJ_BJPG	scenario 3	0.1681 [0.1599,0.1763]
	data18_SCGY_YNQJ_LNSY	scenario 9	0.1682 [0.1594,0.1770]
	data19_SCGY_YNQJ_SXYA	scenario 2	0.1548 [0.1488,0.1609]
	data20_SCGY_YNQJ_FJND	scenario 9	0.1962 [0.1853,0.2071]
	data21_SCGY_YNHH_BJPG	scenario 3	0.5064 [0.4754,0.5373]
	data22_SCGY_YNHH_LNSY	scenario 3	0.3255 [0.3088,0.3423]
	data23_SCGY_YNHH_SXYA	scenario 3	0.3477 [0.3372,0.3582]
	data24_SCGY_YNHH_FJND	scenario 8	0.3052 [0.2927,0.3177]
	data25_SCGY_YNBS_BJPG	scenario 9	0.2498 [0.2424,0.2571]
	data26_SCGY_YNBS_LNSY	scenario 9	0.2969 [0.2855,0.3084]
	data27_SCGY_YNHH_SXYA	scenario 1	0.2907 [0.2828,0.2985]
	data28_SCGY_YNHH_FJND	scenario 9	0.3822 [0.3693,0.3952]

	data29_SCGY_YNPE_BJPG	scenario 8	0.1841 [0.1743,0.1938]
	data30_SCGY_YNPE_LNSY	scenario 8	0.1952 [0.1846,0.2059]
	data31_SCGY_YNBS_SXYA	scenario 8	0.1767 [0.1648,0.1886]
	data32_SCGY_YNPE_FJND	scenario 9	0.2345 [0.2231,0.2459]
Step 2	data01_SCCD_YNQJ_BJPG	scenario 1	0.7434 [0.7359,0.7508]
	data02_SCCD_YNQJ_LNSY	scenario 1	0.7744 [0.7670,0.7818]
	data03_SCCD_YNQJ_SXYA	scenario 1	0.5943 [0.5869,0.6017]
	data04_SCCD_YNQJ_FJND	scenario 1	0.7817 [0.7734,0.7899]
	data05_SCCD_YNHH_BJPG	scenario 1	0.6945 [0.6871,0.7020]
	data06_SCCD_YNHH_LNSY	scenario 1	0.5078 [0.4703,0.5452]
	data07_SCCD_YNHH_SXYA	scenario 1	0.6116 [0.5772,0.6460]
	data08_SCCD_YNHH_FJND	scenario 1	0.5628 [0.5288,0.5967]
	data13_SCCD_YNPE_BJPG	scenario 1	0.8294 [0.8225,0.8363]
	data14_SCCD_YNPE_LNSY	scenario 1	0.8630 [0.8566,0.8695]
	data16_SCCD_YNPE_FJND	scenario 1	0.7772 [0.7699,0.7846]
	data17_SCGY_YNQJ_BJPG	scenario 1	0.6912 [0.6585,0.7240]
	data21_SCGY_YNHH_BJPG	scenario 1	0.6116 [0.5821,0.6412]
	data22_SCGY_YNHH_LNSY	scenario 1	0.3985 [0.3513,0.4457]
	data23_SCGY_YNHH_SXYA	scenario 2	0.6015 [0.5543,0.6487]
Step 3-1	data01_SCCD_YNQJ_BJPG_GDGZ	scenario 2	0.5493 [0.5404,0.5581]
	data02_SCCD_YNQJ_BJPG_GSTS	scenario 3	0.8191 [0.8102,0.8280]
	data03_SCCD_YNQJ_BJPG_GXNN	scenario 2	0.7358 [0.7268,0.7448]
	data04_SCCD_YNQJ_BJPG_QHHD	scenario 1	0.4765 [0.4686,0.4843]
	data05_SCCD_YNQJ_LNSY_GDGZ	scenario 2	0.4830 [0.4743,0.4917]
	data06_SCCD_YNQJ_LNSY_GSTS	scenario 3	0.8382 [0.8300,0.8463]
	data07_SCCD_YNQJ_LNSY_GXNN	scenario 2	0.6282 [0.5653,0.6912]
	data08_SCCD_YNQJ_LNSY_QHHD	scenario 1	0.4465 [0.4385,0.4544]
	data09_SCCD_YNQJ_SXYA_GDGZ	scenario 2	0.4212 [0.4134,0.4289]

data10_SCCD_YNQJ_SXYA_GSTS	scenario 3	0.7432 [0.7331,0.7533]
data11_SCCD_YNQJ_SXYA_GXNN	scenario 2	0.8144 [0.8064,0.8225]
data12_SCCD_YNQJ_SXYA_QHHD	scenario 1	0.5928 [0.5853,0.6003]
data13_SCCD_YNQJ_FJND_GDGZ	scenario 2	0.4128 [0.4051,0.4205]
data14_SCCD_YNQJ_FJND_GSTS	scenario 3	0.8727 [0.8651,0.8804]
data15_SCCD_YNQJ_FJND_GXNN	scenario 2	0.6079 [0.5981,0.6176]
data16_SCCD_YNQJ_FJND_QHHD	scenario 1	0.4374 [0.4294,0.4454]
data17_SCCD_YNHH_BJPG_GDGZ	scenario 2	0.4498 [0.4414,0.4581]
data18_SCCD_YNHH_BJPG_GSTS	scenario 3	0.9207 [0.9146,0.9268]
data19_SCCD_YNHH_BJPG_GXNN	scenario 2	0.8229 [0.8148,0.8310]
data20_SCCD_YNHH_BJPG_QHHD	scenario 1	0.4625 [0.4546,0.4705]
data21_SCCD_YNHH_LNSY_GDGZ	scenario 1	0.3908 [0.3833,0.3984]
data22_SCCD_YNHH_LNSY_GSTS	scenario 3	0.9319 [0.9268,0.9370]
data23_SCCD_YNHH_LNSY_GXNN	scenario 2	0.7995 [0.7909,0.8081]
data24_SCCD_YNHH_LNSY_QHHD	scenario 3	0.3947 [0.3863,0.4031]
data25_SCCD_YNHH_SXYA_GDGZ	scenario 2	0.4137 [0.4054,0.4221]
data26_SCCD_YNHH_SXYA_GSTS	scenario 3	0.8333 [0.8243,0.8422]
data27_SCCD_YNHH_SXYA_GXNN	scenario 2	0.8516 [0.8442,0.8591]
data28_SCCD_YNHH_SXYA_QHHD	scenario 1	0.5289 [0.5213,0.5365]
data29_SCCD_YNHH_FJND_GDGZ	scenario 3	0.3491 [0.3412,0.3570]
data30_SCCD_YNHH_FJND_GSTS	scenario 3	0.9382 [0.9329,0.9434]
data31_SCCD_YNHH_FJND_GXNN	scenario 3	0.4725 [0.4639,0.4811]
data32_SCCD_YNHH_FJND_QHHD	scenario 2	0.7220 [0.7129,0.7311]
data33_SCCD_YNPE_BJPG_GDGZ	scenario 2	0.6788 [0.6698,0.6878]
data34_SCCD_YNPE_BJPG_GSTS	scenario 3	0.7425 [0.7324,0.7525]
data35_SCCD_YNPE_BJPG_GXNN	scenario 2	0.5667 [0.5567,0.5766]
data36_SCCD_YNPE_BJPG_QHHD	scenario 2	0.5002 [0.4910,0.5094]
data37_SCCD_YNPE_LNSY_GDGZ	scenario 2	0.5105 [0.5006,0.5204]
data38_SCCD_YNPE_LNSY_GSTS	scenario 3	0.7188 [0.7083,0.7293]
data39_SCCD_YNPE_LNSY_GXNN	scenario 2	0.5734 [0.5639,0.5830]

	data40_SCCD_YNPE_LNSY_QHHD	scenario 1	0.4401 [0.4321,0.4481]
	data41_SCCD_YNPE_FJND_GDGZ	scenario 2	0.6125 [0.6035,0.6215]
	data42_SCCD_YNPE_FJND_GSTS	scenario 3	0.7373 [0.7258,0.7487]
	data43_SCCD_YNPE_FJND_GXNN	scenario 2	0.4318 [0.4235,0.4402]
	data44_SCCD_YNPE_FJND_QHHD	scenario 1	0.4565 [0.4487,0.4643]
	data45_SCGY_YNQJ_BJPG_GDGZ	scenario 2	0.5319 [0.5231,0.5408]
	data46_SCGY_YNQJ_BJPG_GSTS	scenario 3	0.7886 [0.7799,0.7973]
	data47_SCGY_YNQJ_BJPG_GXNN	scenario 2	0.6692 [0.6598,0.6787]
	data48_SCGY_YNQJ_BJPG_QHHD	scenario 1	0.6087 [0.6010,0.6165]
	data49_SCGY_YNHH_BJPG_GDGZ	scenario 2	0.4930 [0.4848,0.5013]
	data50_SCGY_YNHH_BJPG_GSTS	scenario 3	0.8648 [0.8572,0.8724]
	data51_SCGY_YNHH_BJPG_GXNN	scenario 2	0.7911 [0.7824,0.7998]
	data52_SCGY_YNHH_BJPG_QHHD	scenario 1	0.5094 [0.5014,0.5175]
	data53_SCGY_YNHH_LNSY_GDGZ	scenario 2	0.5462 [0.5364,0.5560]
	data54_SCGY_YNHH_LNSY_GSTS	scenario 3	0.9338 [0.9289,0.9387]
	data55_SCGY_YNHH_LNSY_GXNN	scenario 2	0.7496 [0.7402,0.7589]
	data56_SCGY_YNHH_LNSY_QHHD	scenario 3	0.4822 [0.4737,0.4907]
Step 3-2	data01_SCCD_YNQJ_GDGZ	scenario 1	0.4014 [0.3943,0.4085]
	data02_SCCD_YNQJ_GSTS	scenario 3	0.6017 [0.5934,0.6100]
	data03_SCCD_YNQJ_GXNN	scenario 2	0.5754 [0.5681,0.5827]
	data04_SCCD_YNQJ_QHHD	scenario 1	0.5694 [0.5244,0.6145]
	data05_SCCD_YNHH_GDGZ	scenario 1	0.4178 [0.4105,0.4252]
	data07_SCCD_YNHH_GXNN	scenario 2	0.6371 [0.5909,0.6833]
	data08_SCCD_YNHH_QHHD	scenario 1	0.5210 [0.5136,0.5285]
	data09_SCCD_YNBS_GDGZ	scenario 2	0.5051 [0.4949,0.5153]
	data11_SCCD_YNBS_GXNN	scenario 2	0.5340 [0.5260,0.5419]
	data12_SCCD_YNBS_QHHD	scenario 2	0.5074 [0.4995,0.5152]
	data13_SCCD_YNPE_GDGZ	scenario 2	0.4578 [0.4481,0.4675]
	data14_SCCD_YNPE_GSTS	scenario 1	0.5237 [0.5155,0.5320]

data15_SCCD_YNPE_GXNN	scenario 2	0.6240 [0.6131,0.6349]
data16_SCCD_YNPE_QHHD	scenario 2	0.4673 [0.4592,0.4754]
data17_SCGY_YNQJ_GDGZ	scenario 2	0.4856 [0.4758,0.4955]
data19_SCGY_YNQJ_GXNN	scenario 2	0.5984 [0.5906,0.6063]
data21_SCGY_YNHH_GDGZ	scenario 2	0.4952 [0.4817,0.5087]
data23_SCGY_YNHH_GXNN	scenario 2	0.6038 [0.5961,0.6114]
data24_SCGY_YNHH_QHHD	scenario 1	0.4993 [0.4907,0.5078]
data25_SCGY_YNBS_GDGZ	scenario 2	0.6534 [0.6397,0.6672]
data27_SCGY_YNHH_GXNN	scenario 2	0.5863 [0.5790,0.5936]
data28_SCGY_YNHH_QHHD	scenario 1	0.4515 [0.4443,0.4587]
data29_SCGY_YNPE_GDGZ	scenario 2	0.5378 [0.5298,0.5457]
data31_SCGY_YNBS_GXNN	scenario 2	0.4637 [0.4565,0.4709]
data32_SCGY_YNPE_QHHD	scenario 1	0.3910 [0.3828,0.3993]
data6_SCCD_YNHH_GSTS	scenario 3	0.4811 [0.4256,0.5365]
data10_SCCD_YNBS_GSTS	scenario 3	0.4543 [0.4467,0.4619]
data18_SCGY_YNQJ_GSTS	scenario 3	0.7523 [0.7415,0.7630]
data20_SCGY_YNQJ_QHHD	scenario 3	0.4084 [0.3999,0.4170]
data22_SCGY_YNHH_GSTS	scenario 3	0.6830 [0.6744,0.6915]
data26_SCGY_YNBS_GSTS	scenario 3	0.5352 [0.5259,0.5445]
data30_SCGY_YNPE_GSTS	scenario 3	0.5824 [0.5743,0.5905]

Table S3 Primers used in the study

Gene/locus	Forward primer	Reverse primer	Null allele frequency$
*cox1**	TTGGTCACCCAGAAGTT	ACTATAAAATGGTTTAAGAG	
*nad5**	TTATATCCTTAGAATAAAAACC	TTAGGTTGAGATGGTTTAGG	
GM3-S04#	TTGTGCCAGAAGGACGGATG	GGGCGTCTATTATGCTTTCACT	0.229
GM3-S08#	AGTGCGATGTCTAGCAGTGG	AACCCAGGGCCATTAGCTTC	0.184
GM3-S11#	ACGCCGAGGTATGTATAGTGC	TTGACGGTGCGACTACACAA	0.027
GM3-S13#	GTAATTTGTGGTCCAACGGGC	ACGTTGTCCCGTACAGTCAG	0.135
GM3-S15#	CCAAGTCATATTTGGCGGAGG	GTGTACGGCTACTGGCTTGT	0.246
GM5-S18#	CGCGACACATTCACAATGGA	CACCTGACCTCCAACCCAAA	0.192
GM3-S30#	GCTCCGTCGACATCGCTAAT	GCCTGTGGGTTGGATGAAGA	0.203
GM3-S31#	AGAGATAGCACGCATGACCT	TGTTACTCGCAATCCTCAAGTC	0.102
GM3-S32#	GCTGGCCTTGACTGGAAGAA	GTCTGCAGCCCACCTCAATC	0.201
GM3-S34#	TGAGTTCTCCGATGGCTGTT	TAAGTGTCCTAGCTTGCGCC	0.015
GM3-S41#	TCTAGTGTTTGAAGGGACGCT	GTTGAGCAGCAGGGTCTGAA	0.226
GM3-S64#	TTATTTCAATCGCCGCCTCC	CTCATTGGACGAGCTGAGGT	0.083

* from Wei *et al.* (2015); # from Song *et al.* (2016); $ estimated by Geneland (Guillot *et al.* 2005).

Table S4 Priors used in DIYABC analysis

Step	Parameters	Prior
	N1	10-20000
	N2	10-9000
	N3	10-10000
Step 1	t1	10-10000
	t2	10-10000
	NA	10-20000
	ra	0.01-0.99
	N1	10-30000
	N2	10-10000
Step 2	N3	10-10000
	t1	10-5000
	t2	10-10000
	ra	0.01-0.99
	N1	10-50000
	N2	10-10000
	N3	10-10000
Step 3-1	N4	10-8000
	t1	10-5000
	t2	10-8000
	ra	0.01-0.99
	rb	0.01-0.99
	N1	10-100000
	N2	10-10000
Step 3-2	N3	10-10000
	t1	10-8000
	t2	10-10000
	ra	0.01-0.99

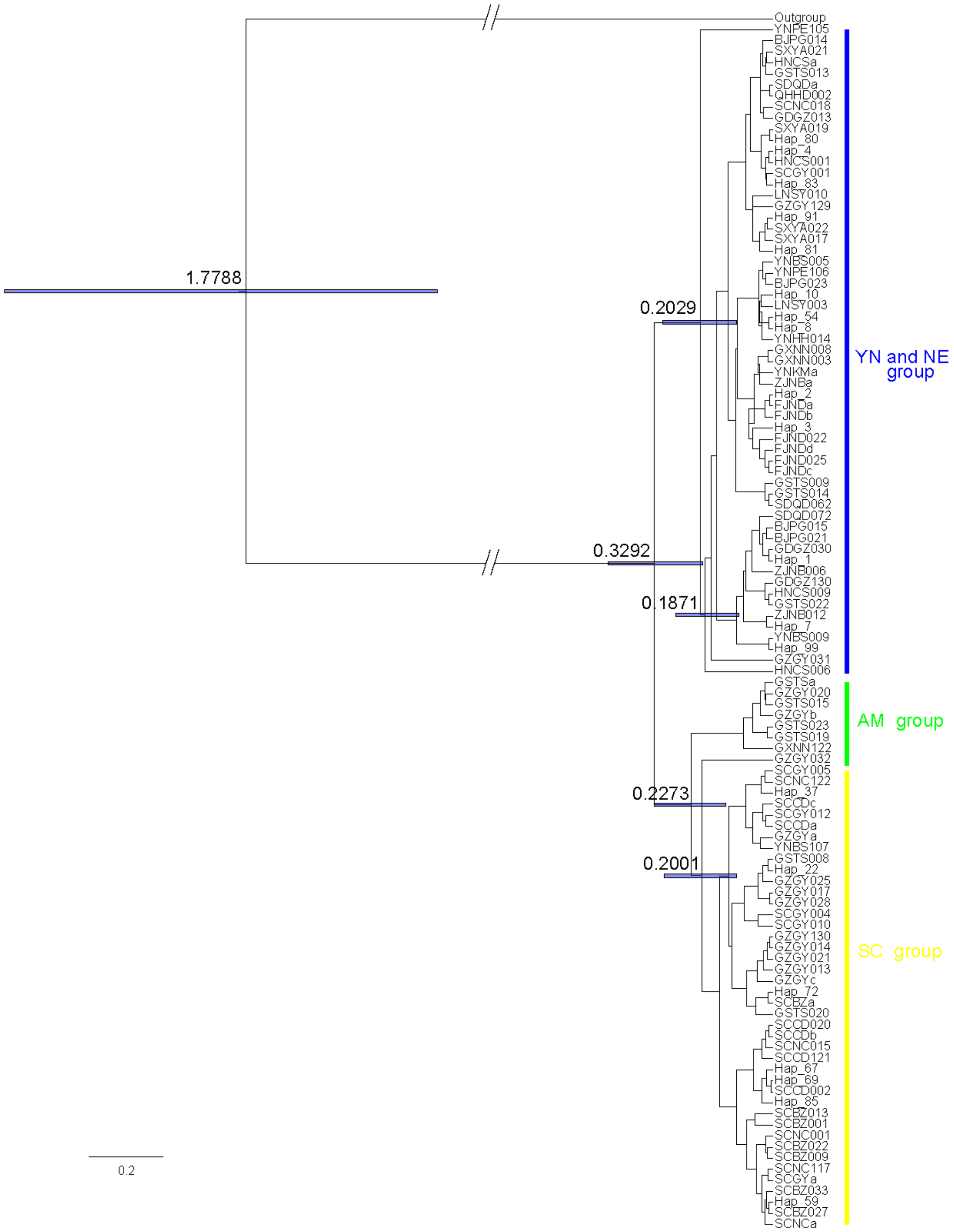

Fig. S1 Dating tree of divergence time for each *Grapholita molesta* group. The divergence time between the major lineages of mitochondrial haplotypes was calculated by BEAST version 1.8.1(Drummond & Rambaut 2007). The *Grapholita dimorpha* was used as out-group (GenBank Number: KJ671625). Based on mitochondrial genes, the divergence time of OFM between populations of Yunnan and Sichuan was dated to 329.2 kya ago, the admixture events occurred at 227.3 kya ago.

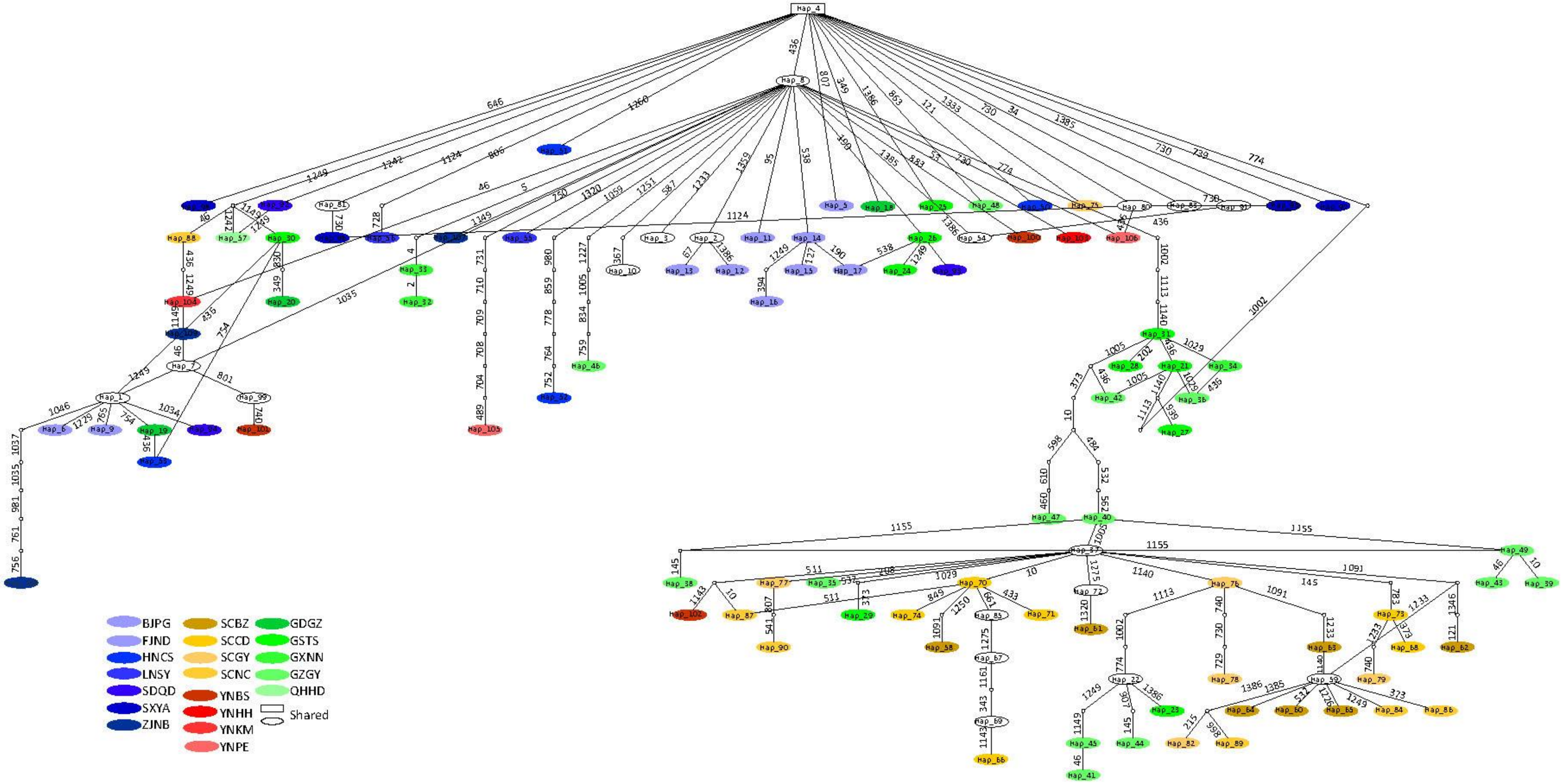

Fig. S2 Statistical parsimony networks of the combined mitochondrial genes. Distribution of each haplotype was listed below.

Hap_1: 57 [BJPG001 BJPG003 BJPG005 BJPG006 BJPG008 BJPG010 BJPG012 BJPG018 BJPG024 FJND014 GDGZ003 GDGZ007 GDGZ010 GDGZ011 GDGZ014 GDGZ015 GDGZ018 GDGZ019 GDGZ020 GDGZ023 GDGZ025 GDGZ026 GDGZ027 GDGZ028 GDGZ031 GDGZ032 GDGZ126 GDGZ127 GDGZ128 GXNN002 GXNN020 GZGY018 HNCS004 HNCS007 HNCS111 LNSY002 LNSY004 LNSY005 LNSY006 LNSY033 LNSY034 LNSY036 LNSY037 LNSY040 LNSY041 LNSY042 LNSY043 LNSY045 SCGY031 SDQD060 SDQD063 SDQD069 SXYA009 SXYA024 YNKM019 YNKM029 YNKM126]

Hap_2: 12 [BJPG002 FJND010 LNSY035 SXYA010 YNBS010 YNBS011 YNBS012 YNBS013 YNBS014 YNBS015 YNBS016 YNPE001]

Hap_3: 4 [BJPG004 BJPG013 SCGY999 SDQD007]

Hap_4: 75 [**BJPG007** BJPG009 BJPG011 BJPG017 BJPG020 **FJND003** FJND004 FJND006 FJND007 FJND011 FJND019 **GSTS003** GSTS005 GSTS012 **GXNN004** GXNN005 GXNN010 GXNN012 GXNN016 GXNN022 **GZGY026 LNSY007** LNSY008 LNSY009 LNSY038 **QHHD001** QHHD003 QHHD004 QHHD005 QHHD006 QHHD007 QHHD008 QHHD009 QHHD013 QHHD015 QHHD018 QHHD019 QHHD020 QHHD021 QHHD022 QHHD023 QHHD024 QHHD025 QHHD026 QHHD027 QHHD028 **SCBZ021** SCBZ023 **SDQD003** SDQD004 SDQD005 SDQD057 SDQD059 SDQD064 SDQD066 SDQD068 SDQD070 **SXYA002** SXYA004 SXYA007 SXYA008 SXYA011 SXYA012 SXYA014 SXYA016 SXYA023 **YNBS007 YNHH001** YNHH015 YNHH016 **YNKM020** YNKM024 **ZJNB002** ZJNB011 ZJNB016]

Hap_5: 1 [BJPG014]

Hap_6: 1 [BJPG015]

Hap_7: 6 [BJPG016 SDQD002 YNKM001 YNKM028 YNKM120 YNKM124]

Hap_8: 106 [BJPG019 FJND008 GDGZ129 GDGZ131 GXNN001 GXNN006 GXNN007 GXNN009 GXNN011 GXNN013 GXNN014 GXNN019 GXNN021 GZGY019 GZGY132 HNCS109 LNSY031 LNSY039 LNSY044 SDQD058 SXYA005 SXYA006 SXYA018 SXYA020 YNBS001 YNBS002 YNBS003 YNBS008 YNBS103 YNBS105 YNBS108 YNBS109 YNBS114 YNBS116 YNHH002 YNHH003 YNHH004 YNHH005 YNHH006 YNHH007 YNHH008 YNHH010 YNHH011 YNHH012 YNHH013 YNHH017 YNHH018 YNHH019 YNHH020 YNKM017 YNKM018 YNKM021 YNKM022 YNKM025 YNKM026 YNKM027 YNKM030 YNKM117 YNKM119 YNKM122 YNKM123 YNKM125 YNPE002 YNPE003 YNPE005 YNPE006 YNPE007 YNPE008 YNPE009 YNPE101 YNPE102 YNPE103 YNPE104 YNPE107 YNPE109 YNQJ001 YNQJ002 YNQJ003 YNQJ004 YNQJ005 YNQJ006 YNQJ007 YNQJ008 YNQJ009 YNQJ010 YNQJ011 YNQJ012 YNQJ013 YNQJ014 YNQJ016 YNQJ017 YNQJ018 YNQJ019 YNQJ020 YNQJ021 YNQJ022 YNQJ023 YNQJ024 ZJNB004 ZJNB005 ZJNB007 ZJNB008 ZJNB010 ZJNB015 ZJNB017 ZJNB018]

Hap_9: 1 [BJPG021]

Hap_10: 2 [BJPG022 SCGY003]

Hap_11: 1 [BJPG023]

Hap_12: 3 [FJND002 FJND012 FJND023]

Hap_13: 2 [FJND005 FJND016]

Hap_14: 4 [FJND009 FJND015 FJND018 FJND021]

Hap_15: 3 [FJND017 FJND020 FJND024]

Hap_16: 1 [FJND022]

Hap_17: 1 [FJND025]

Hap_18: 1 [GDGZ013]

Hap_19: 1 [GDGZ030]

Hap_20: 1 [GDGZ130]

Hap_21: 4 [GSTS001 GSTS004 GSTS007 GSTS017]
Hap_22: 8 [GSTS002 GSTS006 GSTS011 GSTS016 GSTS018 GSTS021 GSTS024 GZGY034]
Hap_23: 1 [GSTS008]
Hap_24: 1 [GSTS009]
Hap_25: 1 [GSTS013]
Hap_26: 1 [GSTS014]
Hap_27: 1 [GSTS015]
Hap_28: 1 [GSTS019]
Hap_29: 1 [GSTS020]
Hap_30: 1 [GSTS022]
Hap_31: 1 [GSTS023]
Hap_32: 1 [GXNN003]
Hap_33: 1 [GXNN008]
Hap_34: 1 [GXNN122]
Hap_35: 2 [GZGY008 GZGY033]
Hap_36: 2 [GZGY010 GZGY027]
Hap_37: 10 [GZGY012 GZGY024 HNCS002 HNCS003 QHHD010 QHHD011 SCNC012 SCNC025 SXYA013 SXYA015]
Hap_38: 1 [GZGY013]
Hap_39: 1 [GZGY014]
Hap_40: 2 [GZGY016 GZGY022]
Hap_41: 1 [GZGY017]
Hap_42: 1 [GZGY020]
Hap_43: 1 [GZGY021]
Hap_44: 1 [GZGY025]
Hap_45: 1 [GZGY028]
Hap_46: 1 [GZGY031]
Hap_47: 1 [GZGY032]
Hap_48: 1 [GZGY129]
Hap_49: 1 [GZGY130]
Hap_50: 1 [HNCS001]
Hap_51: 3 [HNCS005 HNCS008 HNCS110]
Hap_52: 1 [HNCS006]
Hap_53: 1 [HNCS009]
Hap_54: 2 [LNSY001 YNQJ015]
Hap_55: 1 [LNSY003]
Hap_56: 1 [LNSY010]
Hap_57: 1 [QHHD002]
Hap_58: 1 [SCBZ001]
Hap_59: 27 [SCBZ002 SCBZ010 SCBZ024 SCBZ025 SCBZ026 SCBZ028 SCBZ029 SCBZ030 SCBZ031 SCBZ032 SCBZ034 SCBZ132 SCGY028 SCGY029 SCGY038 SCGY039 SCNC003 SCNC005 SCNC006 SCNC017 SCNC019 SCNC021 SCNC024 SCNC118 SCNC120 SCNC121 SCNC124]
Hap_60: 1 [SCBZ009]
Hap_61: 2 [SCBZ011 SCBZ015]
Hap_62: 1 [SCBZ013]
Hap_63: 1 [SCBZ022]

Hap_64: 1 [SCBZ027]
Hap_65: 1 [SCBZ033]
Hap_66: 1 [SCCD002]
Hap_67: 8 [SCCD003 SCCD013 SCCD120 SCGY022 SCGY032 SCGY033 SCGY036 SCNC126]
Hap_68: 3 [SCCD004 SCCD022 SCCD119]
Hap_69: 7 [SCCD005 SCCD008 SCCD023 SCCD118 SCCD124 SCNC014 SCNC026]
Hap_70: 5 [SCCD006 SCCD007 SCCD010 SCCD017 SCCD019]
Hap_71: 1 [SCCD020]
Hap_72: 3 [SCCD021 SCCD122 SCGY026]
Hap_73: 3 [SCCD115 SCCD117 SCCD123]
Hap_74: 1 [SCCD121]
Hap_75: 1 [SCGY001]
Hap_76: 1 [SCGY004]
Hap_77: 1 [SCGY005]
Hap_78: 1 [SCGY010]
Hap_79: 1 [SCGY012]
Hap_80: 2 [SCGY015 SDQD006]
Hap_81: 2 [SCGY021 SXYA003]
Hap_82: 2 [SCGY035 SCGY037]
Hap_83: 2 [SCGY998 YNBS104]
Hap_84: 1 [SCNC001]
Hap_85: 2 [SCNC007 YNPE108]
Hap_86: 2 [SCNC011 SCNC022]
Hap_87: 1 [SCNC015]
Hap_88: 1 [SCNC018]
Hap_89: 1 [SCNC117]
Hap_90: 1 [SCNC122]
Hap_91: 2 [SDQD055 SXYA001]
Hap_92: 2 [SDQD061 SDQD071]
Hap_93: 1 [SDQD062]
Hap_94: 1 [SDQD072]
Hap_95: 1 [SXYA017]
Hap_96: 1 [SXYA019]
Hap_97: 1 [SXYA021]
Hap_98: 1 [SXYA022]
Hap_99: 4 [YNBS004 YNBS113 ZJNB009 ZJNB013]
Hap_100: 1 [YNBS005]
Hap_101: 1 [YNBS009]
Hap_102: 1 [YNBS107]
Hap_103: 1 [YNHH014]
Hap_104: 2 [YNKM023 YNKM121]
Hap_105: 1 [YNPE105]
Hap_106: 1 [YNPE106]
Hap_107: 3 [ZJNB001 ZJNB003 ZJNB020]
Hap_108: 1 [ZJNB006]
Hap_109: 1 [ZJNB012]

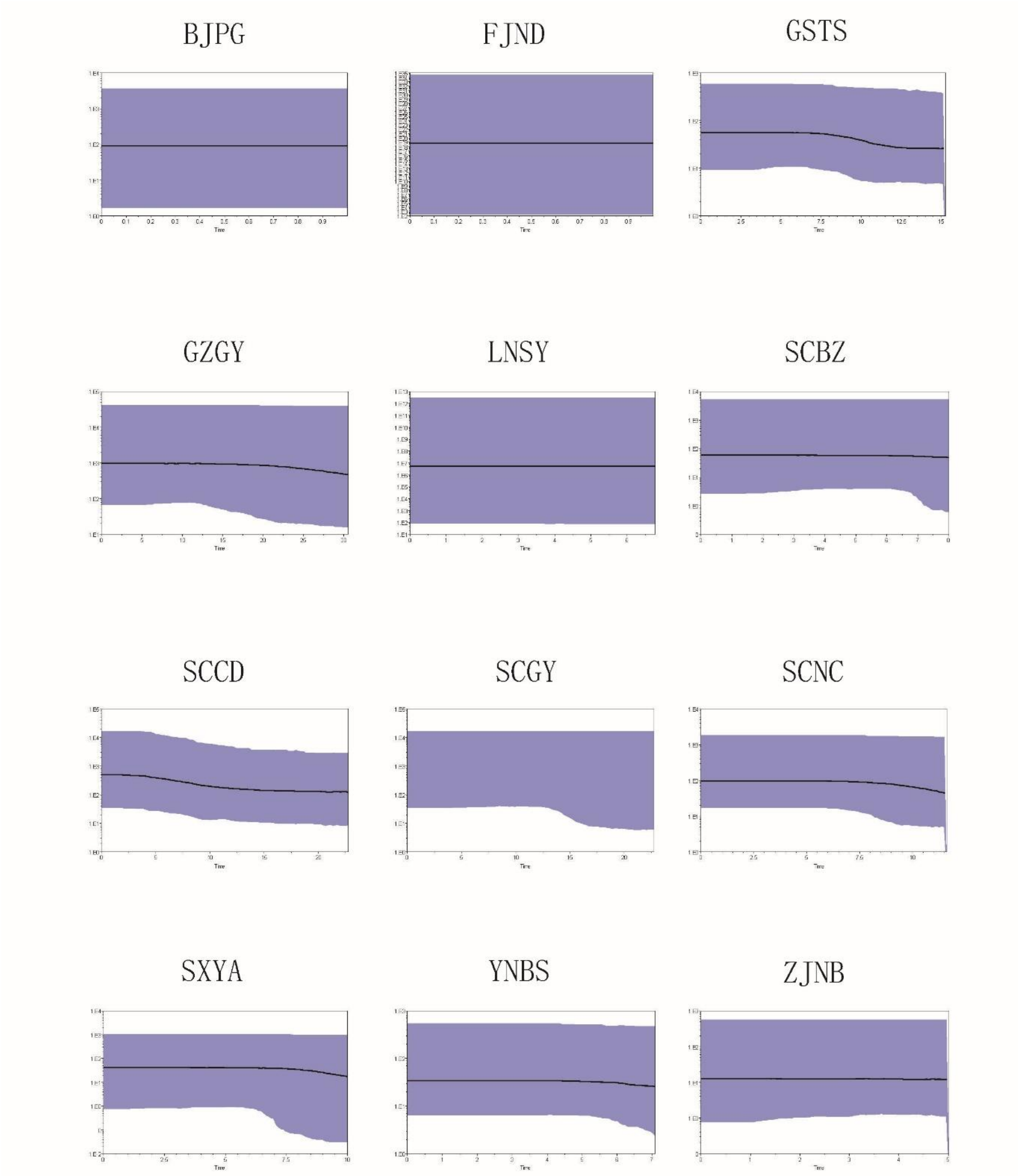

Fig. S3 Bayesian sky plot analysis on the variation in effective population size using BEAST in 12 populations. Other populations were failed to analyze in BEAST.

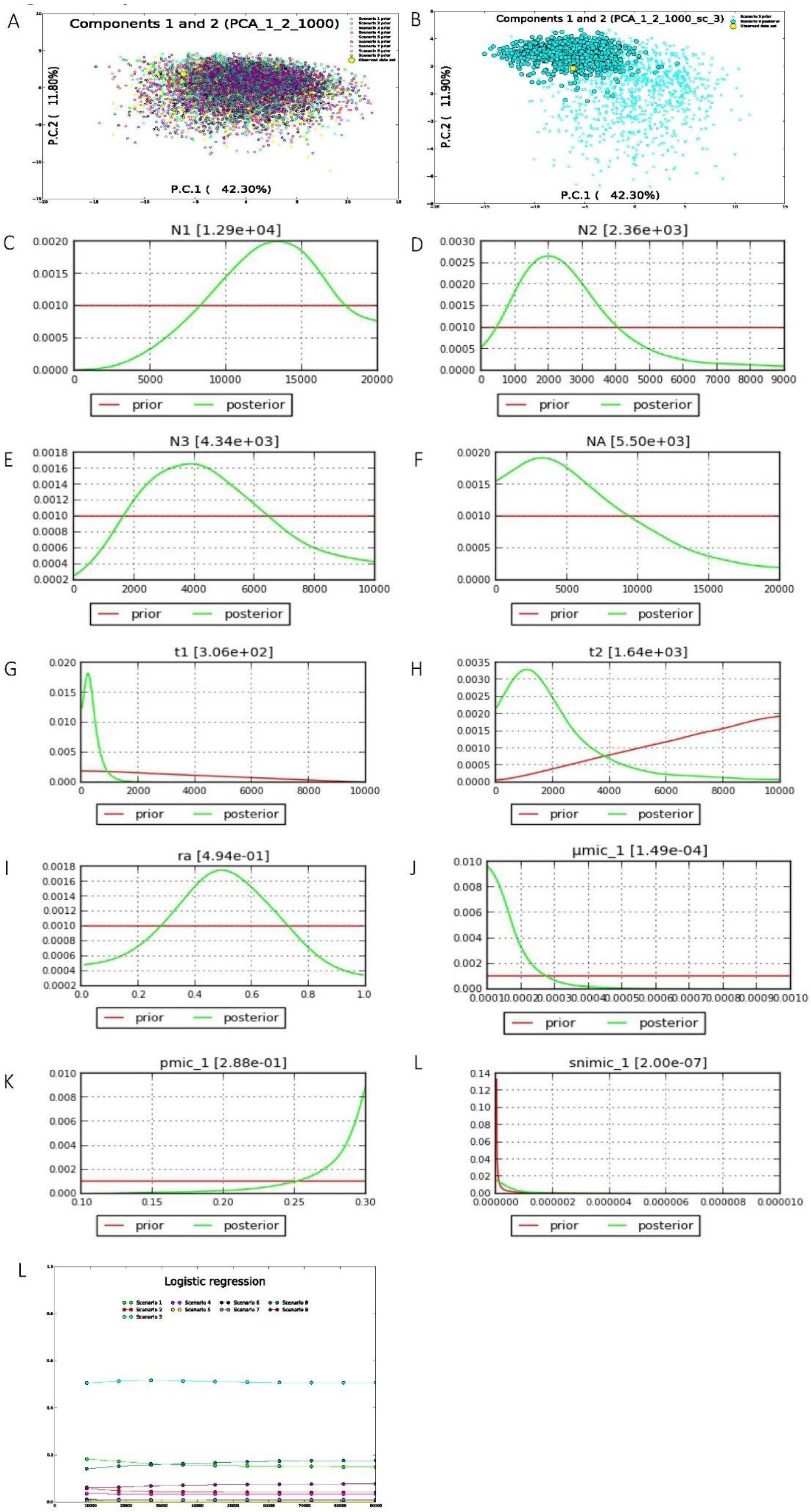
A
Components 1 and 2 (PCA_1_2_1000)
P.C.1 (42.30%)
P.C.2 (11.80%)
B
Components 1 and 2 (PCA_1_2_1000_sc_3)
P.C.1 (42.30%)
P.C.2 (11.90%)
C
N1 [1.29e+04]
D
N2 [2.36e+03]
E
N3 [4.34e+03]
F
NA [5.50e+03]
G
t1 [3.06e+02]
H
t2 [1.64e+03]
I
ra [4.94e-01]
J
µmic_1 [1.49e-04]
K
pmic_1 [2.88e-01]
L
snimic_1 [2.00e-07]
prior
posterior
L
Logistic regression
Scenario 1
Scenario 2
Scenario 3
Scenario 4
Scenario 5
Scenario 6
Scenario 7
Scenario 8
Scenario 9

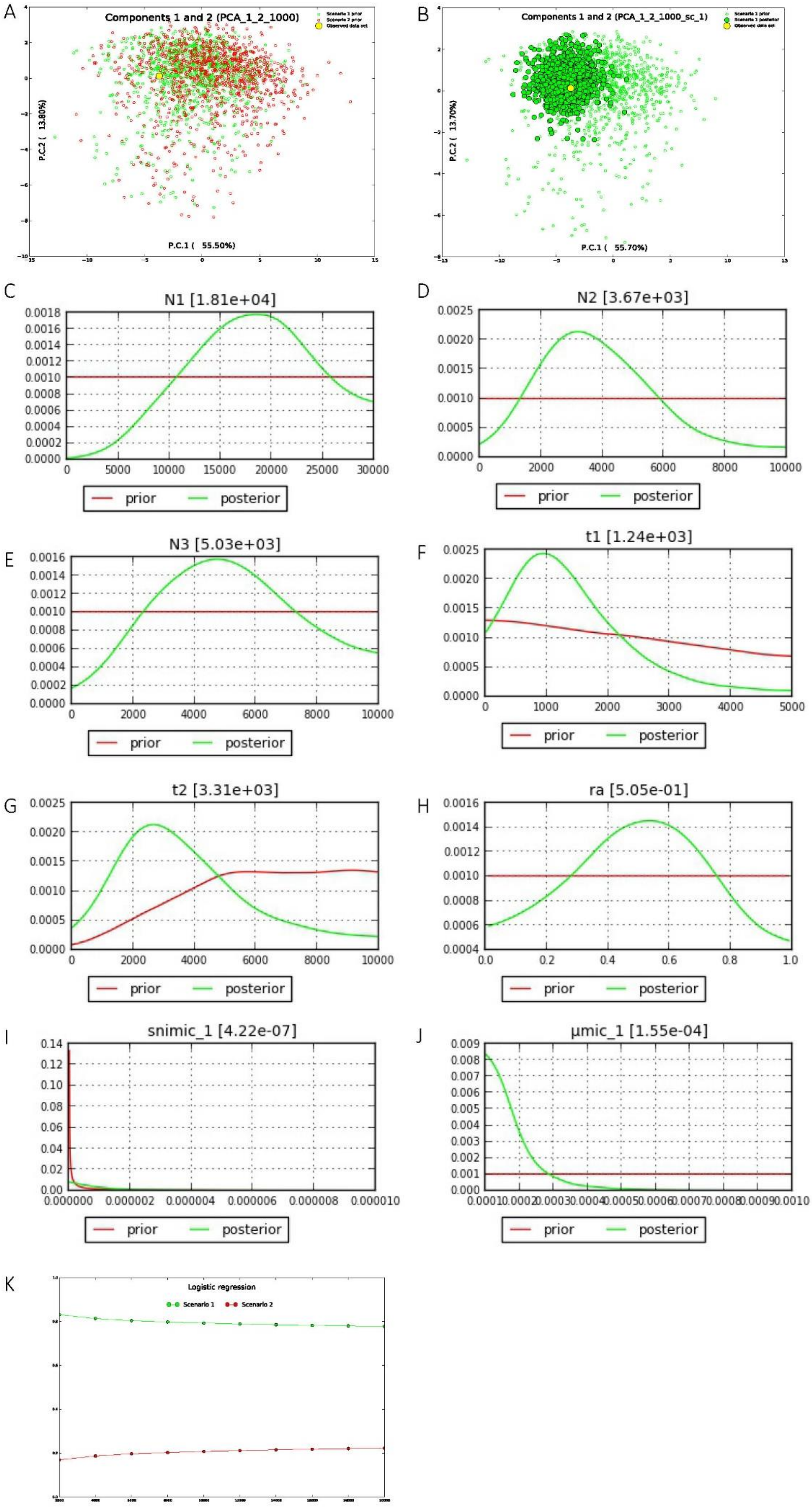
A
Components 1 and 2 (PCA_1_2_1000)
P.C.1 (55.50%)
P.C.2 (13.80%)
B
Components 1 and 2 (PCA_1_2_1000_sc_1)
P.C.1 (55.70%)
P.C.2 (13.70%)
C
N1 [1.81e+04]
D
N2 [3.67e+03]
E
N3 [5.03e+03]
F
t1 [1.24e+03]
G
t2 [3.31e+03]
H
ra [5.05e-01]
I
snimic_1 [4.22e-07]
J
µmic_1 [1.55e-04]
prior
posterior
K
Logistic regression
Scenario 1
Scenario 2

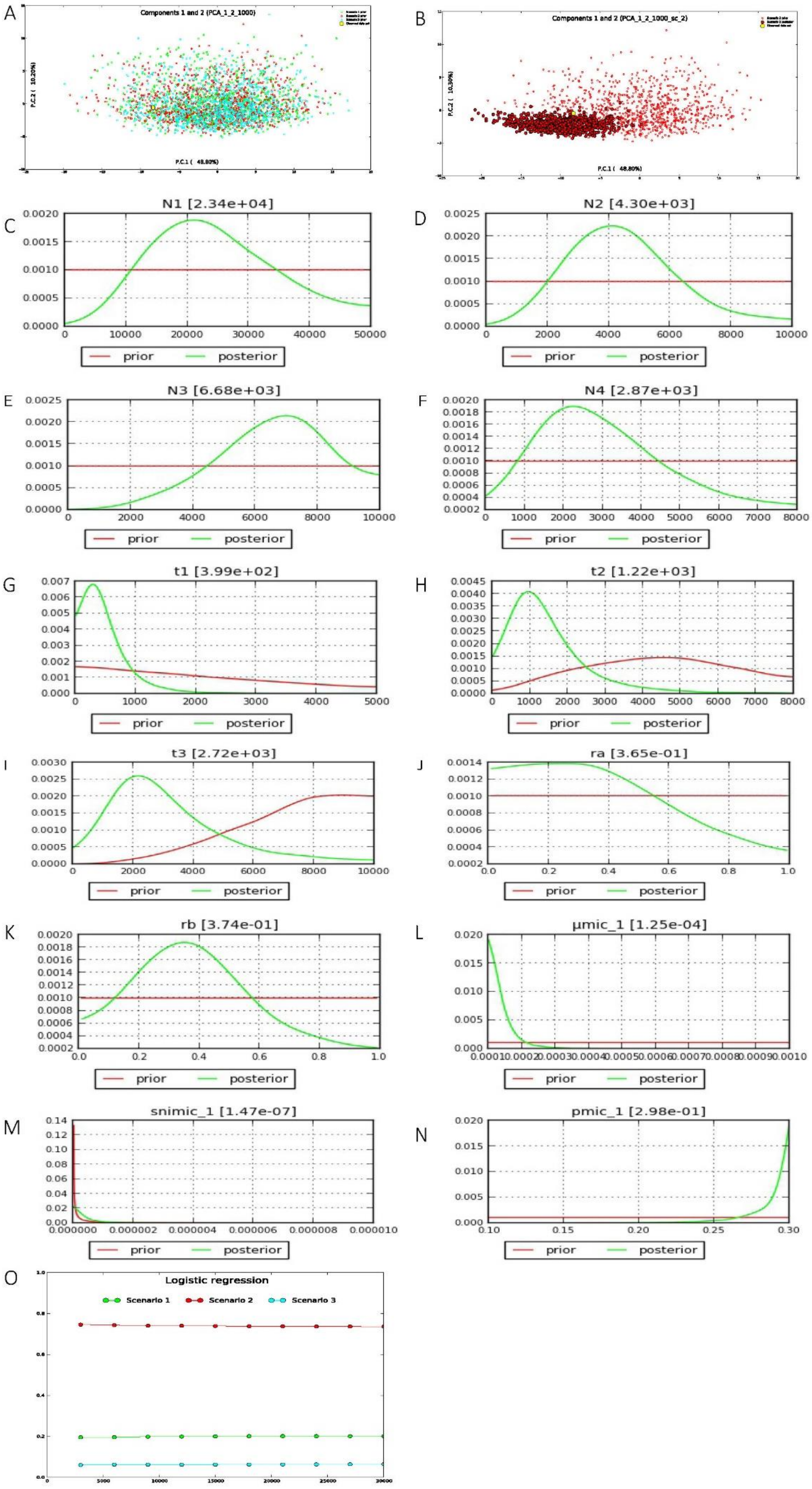
A
Components 1 and 2 (PCA_1_2_1000)
B
Components 1 and 2 (PCA_1_2_1000_sc_2)
C
N1 [2.34e+04]
D
N2 [4.30e+03]
E
N3 [6.68e+03]
F
N4 [2.87e+03]
G
t1 [3.99e+02]
H
t2 [1.22e+03]
I
t3 [2.72e+03]
J
ra [3.65e-01]
K
rb [3.74e-01]
L
µmic_1 [1.25e-04]
M
snimic_1 [1.47e-07]
N
pmic_1 [2.98e-01]
prior
posterior
O
Logistic regression
Scenario 1
Scenario 2
Scenario 3

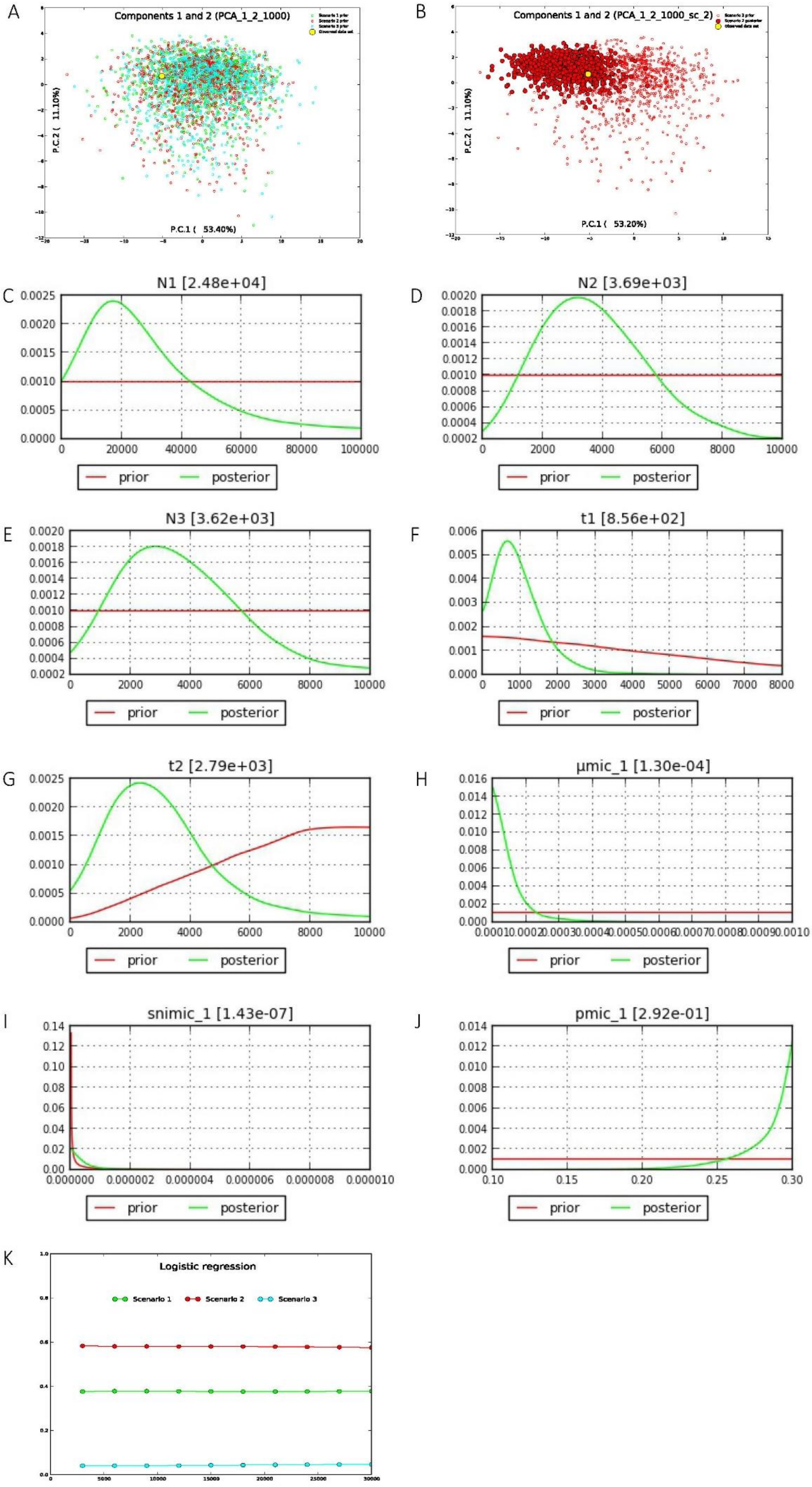
A
Components 1 and 2 (PCA_1_2_1000)
P.C.2 (11.10%)
P.C.1 (53.40%)
B
Components 1 and 2 (PCA_1_2_1000_sc_2)
P.C.2 (11.10%)
P.C.1 (53.20%)
C
N1 [2.48e+04]
D
N2 [3.69e+03]
E
N3 [3.62e+03]
F
t1 [8.56e+02]
G
t2 [2.79e+03]
H
μmic_1 [1.30e-04]
I
snimic_1 [1.43e-07]
J
pmic_1 [2.92e-01]
prior
posterior
K
Logistic regression
Scenario 1
Scenario 2
Scenario 3

Fig. S4 Representative illustration on the pre-evaluation, model checking, posterior distribution and posterior probability in DIYABC analyses. The results were generated based on data set of (SCCD, YNQJ, SXTA), (SCCD, YNQJ, SXTA), (SCCD, YNQJ, BJPG, GXNN) and (SCCD, YNQJ, GXNN), respectively (see Table S2). The four panels show results of step 1, step 2, step 3-1 and step 3-2, respectively. In each panel, figure A and B show the pre-evaluation, model checking, respectively. The last figure shows the posterior probability of each scenario, while the others show the posterior distribution.

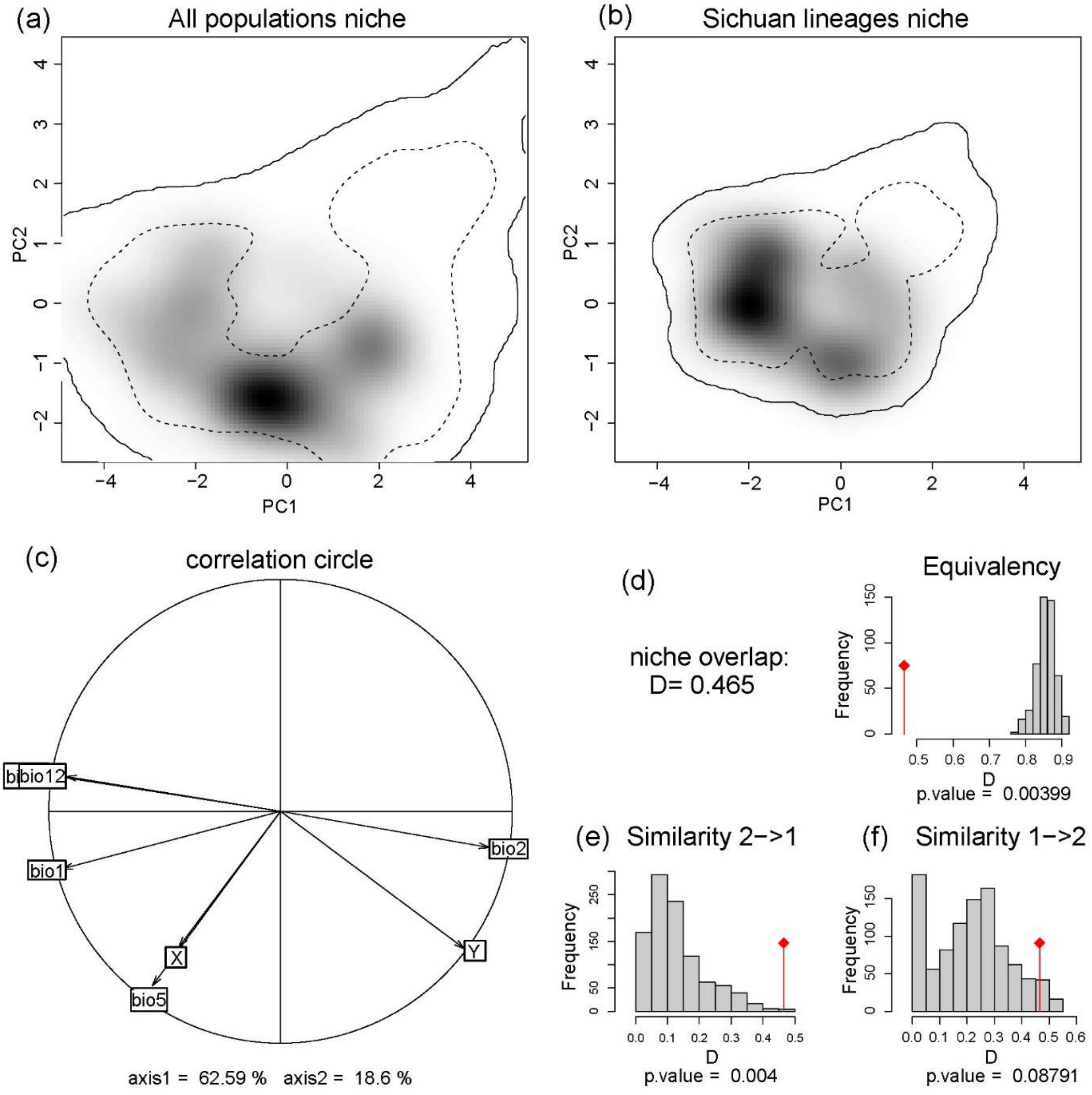

Fig. S5 Niche of *Grapholita molesta* in climatic space based on principal component analysis (PCA-env). (a) Niche of all population of the species along the two first axes of the PCA in the East Asia. (b) Niche of Sichuan lineage. Grey shading shows the density of the occurrences of the species or lineage by cell. The solid and dashed contour lines illustrate 100% and 50% of the available environment, respectively. (c) The contribution of seven bioclimatic variables on the two axes of the PCA and the percentage of inertia explained by the two axes. (d) Niche equivalency test between all populations and Sichuan lineage. Niche similarity test of Sichuan lineage to all populations (e) and all populations to Sichuan lineage (f). For histograms (d)-(f), bars with a diamond represent the position of observed niche overlap D between the two ranges, while grey bars show the simulated niche overlaps. P values of niche equivalency and similarity test were calculated from 500 iterations.

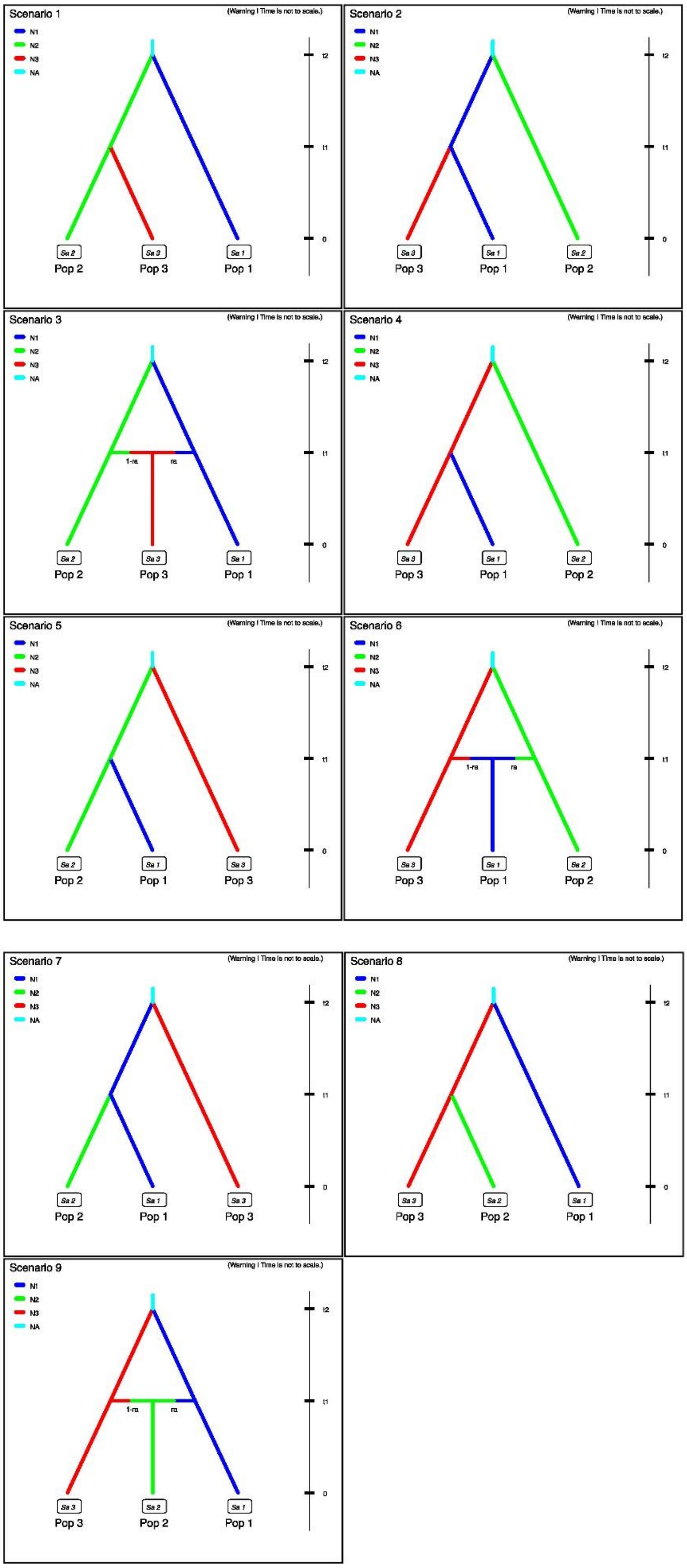

Fig. S6 Scenarios in Step 1 of DIYABC analysis

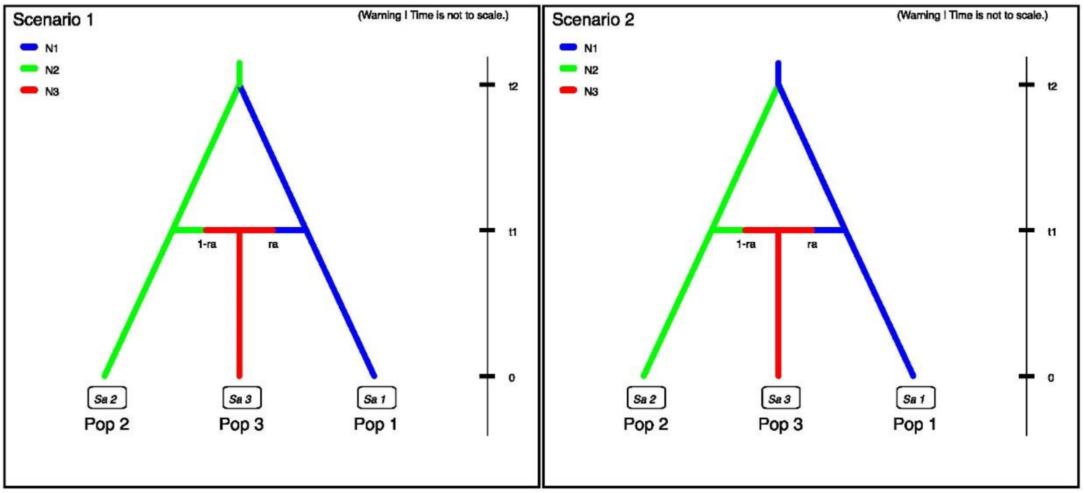

Fig. S7 Scenarios in Step 2 of DIYABC analysis

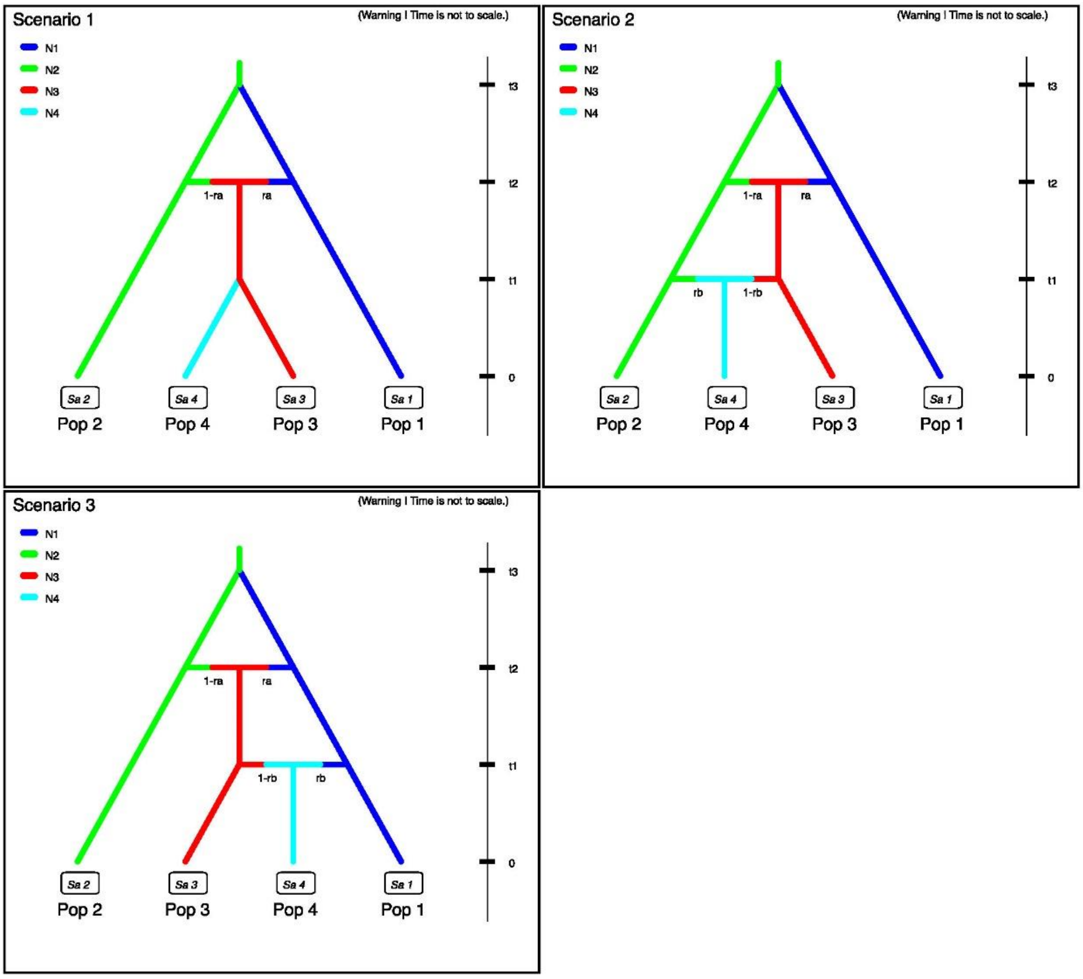

Fig. S8 Scenarios in Step 3-1 of DIYABC analysis

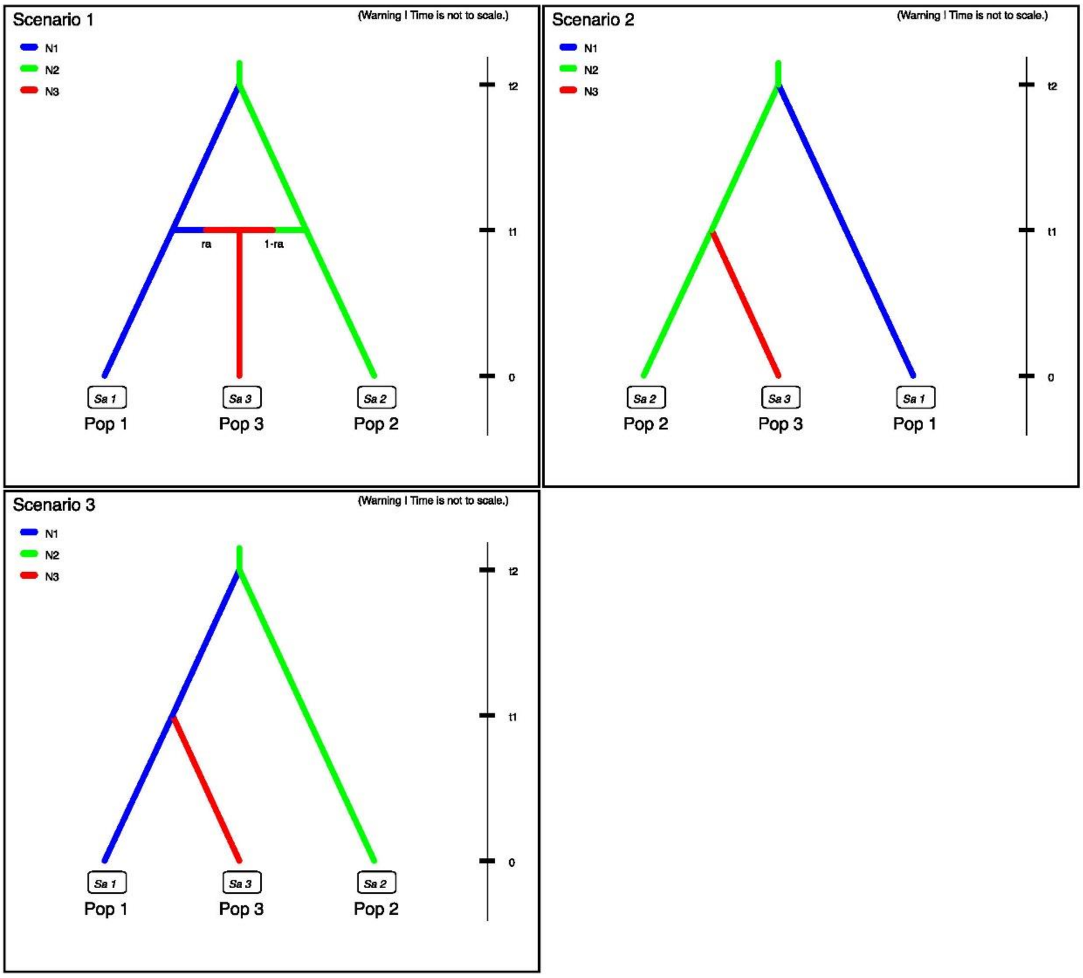

Fig. S9 Scenarios in Step 3-2 of DIYABC analysis

References

Drummond AJ, Rambaut A (2007) BEAST: Bayesian evolutionary analysis by sampling trees. *BMC Evolutionary Biology* **7**, 214.

Guillot G, Mortier F, Estoup A (2005) GENELAND: a computer package for landscape genetics. *Molecular Ecology Notes* **5**, 712-715.

Song W, Cao LJ, Wang YZ, Li BY, Wei SJ (2016) Novel microsatellite markers for the oriental fruit moth Grapholita molesta (Lepidoptera: Tortricidae) and effects of null alleles on population genetics analyses. *Bulletin Entomology Research*, 1-10.

Wei SJ, Cao LJ, Gong YJ, Shi BC, Wang S, Zhang F, Guo XJ, Wang YM, Chen XX (2015) Population genetic structure and approximate Bayesian computation analyses reveal the southern origin and northward dispersal of the oriental fruit moth *Grapholita molesta* (Lepidoptera: Tortricidae) in its native range. *Molecular Ecology* **24**, 4094-4111.
